# Molecular Mechanisms of Emerging Antidepressant Strategies: From Ketamine to Neuromodulation

**DOI:** 10.3390/ijms27010344

**Published:** 2025-12-28

**Authors:** Mateusz Kowalczyk, David Aebisher, Jakub Szpara, Sara Czech, Dorota Bartusik-Aebisher, Gabriela Henrykowska

**Affiliations:** 1 MediPsyche Medical Center, J. Kolinskiego 27, 91-849 Lodz, Poland; mateuszjerzykowalczyk@gmail.com; 2 Department of Photomedicine and Physical Chemistry, Collegium Medicum, Faculty of Medicine, Rzeszów University, 35-310 Rzeszów, Poland; 3 English Division Science Club, Collegium Medicum, Faculty of Medicine, Rzeszów University, 35-310 Rzeszów, Poland; js126214@stud.ur.edu.pl (J.S.); sc126240@stud.ur.edu.pl (S.C.); 4 Department of Biochemistry and General Chemistry, Collegium Medicum, Faculty of Medicine, Rzeszów University, 35-310 Rzeszów, Poland; dbartusikaebisher@ur.edu.pl; 5 Department of Epidemiology and Public Health, Faculty of Medicine, Medical University of Lodz, T. Kosciuszki 4, 90-419 Lodz, Poland

**Keywords:** depression, depressive disorder, molecular mechanisms, novel antidepressant drugs, neuromodulation

## Abstract

Depression is a common, debilitating, and potentially life-threatening mental disorder affecting individuals across all age groups and populations. It represents one of the major challenges of contemporary medicine. It is estimated that more than 300 million people worldwide are affected, and patients with major depressive disorder (MDD) exhibit a significantly increased risk of suicide, underscoring the urgent need for effective and long-lasting therapeutic strategies. Growing evidence indicates that the pathophysiology of depression involves a complex interplay of genetic vulnerability, chronic stress, dysregulation of the hypothalamic–pituitary–adrenal (HPA) axis, neuroinflammation, oxidative stress, mitochondrial dysfunction, and impaired synaptic plasticity, collectively contributing to symptom heterogeneity and treatment resistance. In this review, we synthesize data derived from PubMed, Google Scholar, and ClinicalTrials.gov databases concerning pharmacological and non-pharmacological treatment strategies, with particular emphasis on their cellular and molecular mechanisms of action. We present currently used classes of antidepressant drugs, including selective serotonin reuptake inhibitors (SSRIs), serotonin–norepinephrine reuptake inhibitors (SNRIs), tricyclic antidepressants (TCAs), and monoamine oxidase inhibitors (MAOIs), discussing their limitations in the context of contemporary pathophysiological models of depression. We then focus on emerging therapies targeting the glutamatergic, GABAergic, and dopaminergic systems, including ketamine, esketamine, (R)-ketamine, the dextromethorphan–bupropion combination (DMX–BUP), neurosteroids (zuranolone, brexanolone), as well as selective serotonin receptor modulators (gepirone ER) and dopaminergic modulators (cariprazine). The review is complemented by a discussion of non-pharmacological neuromodulatory approaches, such as transcranial magnetic stimulation (TMS), transcranial direct current stimulation (tDCS), and photobiomodulation. Rather than providing another summary of clinical response indicators, this article integrates the molecular underpinnings of novel antidepressant agents and neuromodulation techniques with current concepts of depression pathophysiology, highlighting their relevance for the development of precise, mechanistically targeted, and multimodal treatment strategies.

## 1. Introduction

### 1.1. Epidemiology

Depression represents one of the most serious and rapidly escalating challenges in contemporary medicine and public health. It is estimated that approximately 4% of the global population has experienced this disorder, including 5.7% of adults, with marked sex-related differences—4.6% among men and 6.9% among women. These differences typically emerge in early adolescence, around 12 years of age, intensify during puberty, and persist into adulthood; nevertheless, depression in men remains a significant and often underrecognized clinical problem [1,2]. Globally, depression affects approximately 322 million individuals, generating a substantial individual, social, and economic burden [3]. The risk of developing depression is strongly modulated by social and economic inequalities, as financial hardship is associated with a significantly higher prevalence of depression irrespective of sex [4]. Particularly concerning trends are observed in children and adolescents. A 2021 review demonstrated that among individuals aged 10–19 years, 8% met the criteria for major depressive disorder (MDD), 4% for dysthymia, while as many as 34% reported severe depressive symptoms. Symptom severity increases markedly after the age of 15, and nearly half of all mental disorders begin before the age of 14. Importantly, the proportion of young individuals with severe depressive symptoms increased from approximately 24% in the period 2001–2010 to 37–38% in 2011–2020 [5]. Data from the second European Health Interview Survey (EHIS-2), encompassing 27 European countries (n = 258,888), further indicate a higher prevalence of depression among older adults, individuals with chronic diseases or limited mobility, those with extreme body mass index (BMI) values, daily smokers, physically inactive individuals, and those with lower income and educational levels. Prevalence rates ranged from 2.56% in Slovakia to 10.33% in Iceland [6]. Collectively, these data underscore the magnitude of the problem and the pronounced population heterogeneity of depression, highlighting the urgent need for more effective and more precise therapeutic strategies.

### 1.2. Risk Factors

Depression is a disorder with a highly complex etiology, encompassing a broad spectrum of psychological, social, and biological factors. As aptly summarized by C. Hammen, cognitive vulnerabilities—such as negative thinking patterns and a propensity for rumination—play a significant role by increasing the risk of recurrent depressive episodes. Stress remains a key precipitating factor, as depressive episodes are frequently preceded by stressful life events, while early childhood trauma markedly enhances stress sensitivity later in life. Family burden also constitutes an important element, including genetic predisposition, limited emotional support, and the reinforcement of maladaptive relational patterns. Interpersonal relationships and the social environment exert a substantial influence, as chronic conflict, sustained tension, and weakened social bonds increase the risk of depression, particularly during adolescence and early adulthood. Personality traits such as high neuroticism and anhedonia are likewise associated with increased vulnerability to disease development. Sex-related differences indicate that women experience depression more frequently, which may be attributable to greater exposure to violence and social inequalities, caregiving burdens, and a stronger tendency toward ruminative coping strategies [7]. Beyond psychosocial determinants, the literature also identifies numerous somatic and psychiatric risk factors, including occupational difficulties, anxiety and personality disorders, alcohol dependence, anemia, autoimmune diseases, cardiovascular disorders, thyroid dysfunction, and rheumatoid arthritis [8]. Notably, these diverse risk factors converge on shared biological pathways related to stress responsivity, immune system activation, disturbances in oxidative balance, and impaired neuronal plasticity.

### 1.3. Patomechanism of Depression

Heritability plays a significant role in the development of depression. Family and twin studies indicate that the heritability of MDD is approximately 31–42%, which is consistent with the prevailing hypothesis emphasizing the critical importance of gene-environment interactions in the etiology of the disorder [9]. From a neurobiological perspective, depression is increasingly viewed not as a dysfunction of a single neurotransmitter system, but rather as a consequence of dysregulation within interconnected molecular and cellular networks responsible for stress adaptation, neuroplasticity, energy metabolism, and immune signaling.

Serotonin (5-hydroxytryptamine, 5-HT) and its receptors play a crucial role in the regulation of mood, stress reactivity, and cognitive functions. In particular, the 5-HT1A receptor is involved in the modulation of depressive symptoms and stress responses, whereas the 5-HT2A and 5-HT2C receptors are implicated in the pathology of mood disorders and neurodegenerative processes. In addition, serotonin interacts with orexinergic neurons in the lateral hypothalamus, thereby influencing emotional regulation and stress responses [10,11]. However, accumulating evidence suggests that monoaminergic disturbances alone are insufficient to explain the complex pathophysiology of depression, and that mechanisms related to synaptic plasticity play a central role. Brain-derived neurotrophic factor (BDNF) supports neuronal survival, growth, and differentiation, and its reduced levels—observed in patients with depression—correlate with impaired synaptic plasticity, astrocytic and microglial dysfunction, and disrupted neuronal communication [12].

**Figure 1:**
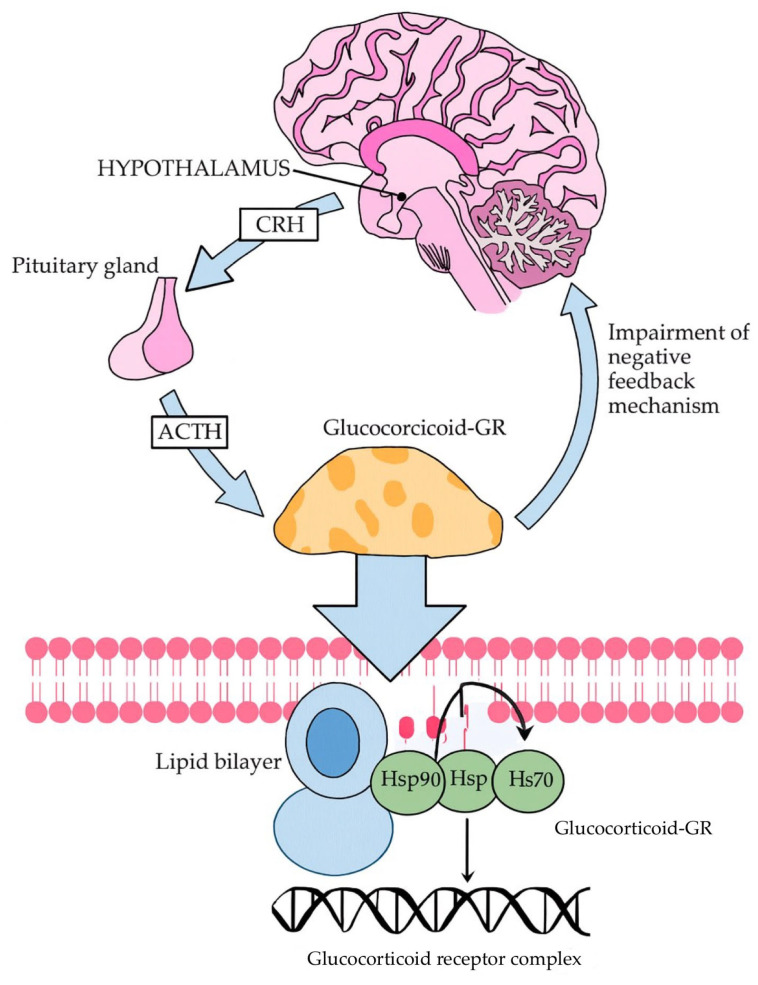
Schematic representation of HPA axis function and intracellular glucocorticoid receptor signaling.

A central component of the biological stress response is the hypothalamic–pituitary–adrenal (HPA) axis, whose chronic hyperactivation constitutes one of the most well-documented pathogenetic mechanisms of depression. The HPA axis mediates the hormonal response to stress, which is a major precipitating factor for depressive episodes. Chronic stress leads to increased secretion of corticotropin-releasing hormone (CRH), adrenocorticotropic hormone (ACTH), and arginine vasopressin (AVP), resulting in sustained stimulation of the adrenal cortex and excessive exposure of the brain to glucocorticoids. As illustrated in Figure 1, persistently elevated glucocorticoid levels disrupt the negative feedback mechanism at the level of glucocorticoid receptors (GR), thereby preventing effective inhibition of the HPA axis. At the cellular level, glucocorticoids diffuse across the lipid bilayer and bind to cytoplasmic GRs associated with the chaperone proteins Hsp90 and Hsp70, forming an activated complex that translocates to the nucleus and regulates gene expression. Dysregulation of this pathway leads to hippocampal neuronal atrophy, impaired neuroplasticity, and increased vulnerability to the development of depression [13,14].

An increasing body of evidence also points to a significant contribution of inflammatory processes to the pathogenesis of MDD. Patients with depression exhibit reduced serum zinc levels, activation of T lymphocytes, and elevated concentrations of acute-phase proteins and proinflammatory cytokines [15,16,17]. These observations are complemented by metabolomic studies, which are gaining importance as a tool for analyzing the complex biochemical alterations accompanying depression. The application of techniques such as proton nuclear magnetic resonance spectroscopy (^1^H NMR), gas chromatography–mass spectrometry (GC-MS), and liquid chromatography–mass spectrometry (LC-MS) has revealed disturbances in amino acid, carbohydrate, and lipid metabolism, as well as in energy-related and tryptophan pathways. Metabolomics enables not only discrimination between healthy and affected individuals but also the assessment of therapeutic intervention efficacy [18].

Oxidative stress (OS) represents another key component of depression pathophysiology. Reactive oxygen species (ROS), which under physiological conditions serve signaling functions, become harmful when their production exceeds the antioxidant capacity of the organism. This imbalance leads to lipid peroxidation, protein and DNA damage, activation of inflammatory pathways, apoptosis, and neurodegeneration. The brain is particularly vulnerable to OS due to its high oxygen consumption, lipid-rich composition, and relatively limited antioxidant defenses. In MDD, increased levels of oxidative stress markers—such as malondialdehyde (MDA) and 8-hydroxy-2-deoxyguanosine (8-OHdG)—telomere shortening, dysregulated nitric oxide signaling, cyclooxygenase-2 (COX-2) activation, and elevated concentrations of proinflammatory cytokines (IL-1, IL-6, TNF-α) have been reported. Concurrently, reduced levels of non-enzymatic antioxidants and impaired activity of antioxidant enzymes are observed, further exacerbating OS and inflammation [19].

Growing attention has also been directed toward mitochondrial dysfunction as a central mechanism underlying depression. In both patients with MDD and animal models, abnormalities in mitochondrial morphology—including swelling, disrupted cristae structure, fragmentation, and altered distribution—have been observed in neurons of the prefrontal cortex and hippocampus. These alterations are closely associated with depression-like behaviors. At the functional level, impaired mitochondrial energy metabolism is evident, characterized by reduced glucose metabolism, decreased ATP production, and diminished activity of respiratory chain complexes (I–V). At the same time, increased anaerobic glycolysis and elevated lactate levels suggest the presence of compensatory mechanisms. Mitochondrial quality control processes, including mitochondrial biogenesis and mitophagy, are also disrupted, leading to the accumulation of damaged organelles and increased neuronal vulnerability to stress [20].

At the intersection of many of these mechanisms lies the signaling pathway of mechanistic target of rapamycin (mTOR) kinase, which plays a pivotal role in regulating synaptic protein synthesis, neuronal survival, and synaptic plasticity. In patients with MDD, mTOR activity- particularly in the prefrontal cortex and amygdala—is significantly reduced, resulting in decreased expression of synaptic proteins such as PSD-95, GluR1, and GluR2, as well as impaired synaptogenesis. mTOR functions within two distinct complexes: mTORC1, which responds to metabolic cues and oxidative stress, and mTORC2, which regulates cytoskeletal organization and Akt/PKB activation. Inhibition of mTORC1 activity in depression limits local protein synthesis at synapses and weakens neuronal adaptive capacity to stress [21].

Glycogen synthase kinase 3β (GSK-3β) represents another critical regulatory hub integrating multiple signaling pathways involved in depression pathogenesis. In patients with MDD, elevated GSK-3β activity has been observed, particularly in the prefrontal cortex. Preclinical studies demonstrate that overexpression or constitutive activation of this kinase enhances depressive- and anxiety-like behaviors, whereas its partial inhibition produces antidepressant-like effects. GSK-3β is inhibited by the PI3K/Akt pathway and BDNF–TrkB signaling and activated by 5-HT_2_A and dopamine D2 receptors, N-methyl-D-aspartate receptors (NMDARs), and phosphatases. Excessive GSK-3β activity promotes long-term synaptic depression (LTD), dendritic retraction, AMPA receptor internalization, and impaired synaptic plasticity in the prefrontal cortex and hippocampus. Moreover, this kinase enhances inflammatory responses and influences epigenetic regulation, thereby linking neurotrophic, inflammatory, and stress-related disturbances into a coherent pathogenic mechanism [22].

### 1.4. Aim and Scope of the Review

Despite substantial advances in research on the neurobiology of depression and the rapid development of novel therapeutic strategies, the available literature largely remains fragmented, focusing either on classical antidepressant drugs or on selected, isolated molecular pathways. At the same time, an increasing body of evidence indicates that effective antidepressant interventions—both pharmacological and neuromodulatory—converge on shared cellular mechanisms regulating synaptic plasticity, stress adaptation, energy metabolism, and inflammatory responses. However, comprehensive reviews integrating these strategies within a single, coherent pathophysiological model of depression are still lacking.

The aim of this review is to provide a synthetic, mechanistic perspective on contemporary strategies for the treatment of depression, taking into account both the limitations of conventional therapies and the biological foundations of emerging interventions. In contrast to reviews that primarily focus on comparing the clinical efficacy of individual modalities, this article emphasizes the identification of common signaling nodes that represent points of convergence for different forms of therapy. Such an approach enables a deeper understanding of how interventions of ostensibly distinct nature—pharmacological and neuromodulatory—can produce convergent biological effects at the cellular and molecular levels.

The novelty of this review lies in the integration of data on classical and novel antidepressant drugs with current concepts of neuromodulation in the context of shared pathogenic mechanisms of depression. By juxtaposing these strategies within a unified mechanistic framework, this work provides a rationale for the development of precise, mechanistically targeted, and multimodal therapeutic approaches that address the biological and clinical heterogeneity of depression.

## 2. Molecular Mechanisms and Clinical Status of Emerging Therapeutic Strategies

### 2.1. Classical Antidepressant Medications

#### 2.1.1. Selective Serotonin Reuptake Inhibitors (SSRIs)

SSRIs constitute the cornerstone of pharmacotherapy for depression, and their primary mechanism of action involves inhibition of the serotonin transporter (SERT), leading to increased availability of 5-HT in the synaptic cleft and enhanced signaling through postsynaptic serotonin receptors. Long-term administration of SSRIs induces neuroadaptive processes, including the gradual desensitization of presynaptic 5-HT_1_A and 5-HT_1_B autoreceptors, resulting in a sustained increase in serotonin release and stabilization of serotonergic neurotransmission. Secondary activation of intracellular cascades follows, including cAMP-dependent pathways, protein kinase A (PKA), and the transcription factor CREB, ultimately leading to changes in the expression of genes involved in synaptic plasticity, neurogenesis, and the functioning of limbic circuits implicated in mood regulation [23].

Clinical data confirm the efficacy of SSRIs in the treatment of depression, with meta-analyses indicating that initial therapeutic effects may emerge as early as the first week of treatment, challenging the traditional notion that several weeks are required before a clinical response becomes apparent [24]. Moreover, SSRIs have been shown to rapidly modulate emotional information processing, leading to a reduction in negative cognitive biases through improved recognition of positive emotions and social cues, which may precede full improvement of affective symptoms [25,26]. In pediatric and adolescent populations, although psychotherapy remains the first-line treatment, SSRIs—particularly fluoxetine—have demonstrated efficacy in moderate to severe depressive episodes and in patients who do not respond adequately to psychotherapeutic interventions [27].

Despite their well-established clinical position, the use of SSRIs is associated with significant limitations. The most commonly observed adverse effects include early exacerbation of anxiety and agitation, sexual dysfunction, sleep disturbances, and gastrointestinal symptoms, all of which may negatively affect treatment adherence. Furthermore, particularly in pediatric populations, the long-term safety of SSRI use remains incompletely characterized. Limited efficacy in a subset of patients, delayed attainment of full therapeutic effects, and the adverse-effect profile collectively underscore the need for the development of novel treatment strategies for depression that extend beyond classical modulation of the monoaminergic system [28].

#### 2.1.2. Serotonin–Norepinephrine Reuptake Inhibitors (SNRIs)

SNRIs exert their antidepressant effects through the simultaneous inhibition of SERT and the norepinephrine transporter (NET), resulting in increased concentrations of both monoamines within the synaptic cleft and enhanced as well as prolonged signaling at postsynaptic receptors. This mechanism leads to modulation of neuronal circuits involved in mood regulation, emotional reactivity, and stress processing. Currently, five agents from this class have been approved by the U.S. Food and Drug Administration (FDA): desvenlafaxine, duloxetine, levomilnacipran, milnacipran, and venlafaxine. Of particular clinical relevance are duloxetine, which is considered a first-line therapy for MDD and remains one of the most frequently prescribed agents for this condition, and venlafaxine, which—beyond its effects on serotonergic and noradrenergic systems—also inhibits dopamine reuptake, with well-documented efficacy in the treatment of MDD [29].

Beyond their classical effects on monoaminergic neurotransmission, chronic SNRI therapy induces additional molecular effects of potential pathophysiological relevance. Preclinical studies have demonstrated that long-term administration of SNRIs increases the expression of connexin 43 (Cx43) in the prefrontal cortex, thereby preventing chronic stress–induced astrocytic gap junction dysfunction. This effect was associated with the reversal of depression-like behaviors induced by pharmacological blockade of gap junctions, highlighting the significant contribution of glial cells and astrocytic communication to the mechanisms of action of SNRIs [30]. These observations suggest that the antidepressant effects of this drug class may extend beyond direct modulation of neurotransmission and involve regulation of neuron–glia interactions.

Despite their documented clinical efficacy, the use of SNRIs is associated with notable safety limitations. The most commonly reported adverse effects include nausea, dizziness, excessive sweating, insomnia, anxiety, constipation, urinary retention, decreased appetite, headache, and sexual dysfunction. In addition, agents from this class may increase the risk of serotonin syndrome, induce elevations in blood pressure, and, less frequently, cause hepatotoxicity. Serotonin syndrome has remained one of the principal clinical limitations of SNRI therapy for many years, underscoring the need for further research aimed at improving safety profiles and developing more selective and better-tolerated therapeutic strategies [31].

#### 2.1.3. Tricyclic Antidepressants (TCAs)

TCAs have been used in the treatment of depression since the 1950s, when they were first approved for the management of mood disorders [32]. The most commonly used representatives of this class include imipramine, amitriptyline, clomipramine, desipramine, nortriptyline, and doxepin. The primary mechanism of action of TCAs involves inhibition of the presynaptic reuptake of serotonin and norepinephrine through blockade of their respective membrane transporters, resulting in increased concentrations of both monoamines in the synaptic cleft and enhanced signaling at postsynaptic receptors. The augmented monoaminergic neurotransmission subsequently initiates secondary, long-term adaptive processes within the central nervous system, including changes in receptor sensitivity and expression, regulation of intracellular signaling pathways, and modulation of neuroplasticity, which collectively underlie the sustained antidepressant effect observed during chronic treatment [33].

Individual TCAs differ in their pharmacodynamic profiles. Amitriptyline, imipramine, and desipramine exhibit strong inhibition of serotonin reuptake, whereas clomipramine preferentially blocks norepinephrine reuptake. Nortriptyline affects both serotonergic and noradrenergic systems and additionally exerts pronounced central anticholinergic effects [34,35]. Beyond direct modulation of monoamine transporters, TCAs also exert downstream effects, including antagonism of 5-HT_2_A and 5-HT_2_C serotonin receptors, which promotes increased release of norepinephrine and dopamine in cortical regions. Moreover, some TCAs—particularly amitriptyline—have been shown to increase the expression of connexin 43 (Cx43), thereby enhancing astrocytic gap junction communication. This mechanism may contribute to the alleviation of depressive symptoms and underscores the relevance of glial mechanisms in the actions of this drug class [36,37].

Despite their documented clinical efficacy, the use of TCAs is associated with significant safety and tolerability limitations. A key concern is their receptor non-selectivity, including blockade of muscarinic, histaminergic, and α_1_-adrenergic receptors. Anticholinergic adverse effects—such as dry mouth, constipation, accommodation disturbances, and urinary retention—are common and substantially limit the use of TCAs, particularly in elderly patients. The adverse-effect profile and risk of toxicity, especially in cases of overdose, have led to TCAs being less frequently employed as first-line agents in the treatment of MDD, despite their preserved antidepressant efficacy [38].

#### 2.1.4. Monoamine Oxidase Inhibitors (MAOIs)

MAOIs, such as phenelzine, tranylcypromine, and isocarboxazid, belong to the earliest developed classes of antidepressant drugs and exert their effects through irreversible binding and inactivation of monoamine oxidase type A and B enzymes (MAO-A and MAO-B). These enzymes are located primarily in the mitochondria of presynaptic nerve terminals and in glial cells, where they are responsible for the oxidative deamination of monoamines. Inhibition of MAO-A activity limits the catabolism of serotonin and norepinephrine, whereas blockade of MAO-B reduces dopamine degradation, resulting in a marked increase in intra-neuronal and synaptic concentrations of these neurotransmitters. Chronic monoamine oxidase inhibition subsequently induces secondary, long-term neuroadaptive processes driven by sustained enhancement of monoaminergic signaling, including regulation of monoamine receptor sensitivity and expression, activation of intracellular signaling pathways related to synaptic plasticity, and increased expression of neurotrophic factors. These changes promote durable normalization of neuronal circuit function involved in mood regulation and emotional behavior [39].

The clinical efficacy of MAOIs has been confirmed in observational and retrospective studies, particularly in patients with severe, atypical, or treatment-resistant forms of depression. In a retrospective study including 400 patients, 56% achieved ratings of “much improved” or “very much improved” on the Clinical Global Impression–Improvement (CGI-I) scale, supporting the high effectiveness of this drug class in selected clinical populations [40].

Despite their documented antidepressant efficacy, the use of MAOIs is associated with significant safety and tolerability limitations. The most commonly reported adverse effects include nausea, dry mouth, constipation or diarrhea, sleep disturbances (insomnia or hypersomnia), dizziness, and a sense of lightheadedness. In the case of transdermal formulations, local allergic skin reactions have also been observed [41]. A major clinical limitation of this drug class is the risk of severe drug–drug and drug-food interactions resulting from non-selective and irreversible monoamine oxidase inhibition, which necessitates strict therapeutic monitoring and substantially restricts routine clinical use. Consequently, MAOIs are currently employed primarily in selected cases of treatment-resistant depression, despite their preserved high pharmacological efficacy. Table 1 summarizes classical antidepressant agents together with a concise overview of their molecular mechanisms, synaptic effects, and downstream pathways.

### 2.2. New Targets in Depression Therapy

#### 2.2.1. Ketamine

In recent years, ketamine has emerged as one of the most important and best-documented novel pharmacological interventions in the treatment of depression, particularly in patients with treatment-resistant depression (TRD). It is a non-competitive antagonist of N-methyl-D-aspartate receptors (NMDARs) and has remained in routine clinical use as an anesthetic agent for more than five decades [42]. Its rapid and pronounced antidepressant effects have challenged the long-standing monoaminergic paradigm and shifted scientific interest toward glutamatergic mechanisms and synaptic neuronal plasticity.

At the molecular level, ketamine blocks NMDARs located predominantly on GABAergic interneurons, leading to their functional silencing and secondary disinhibition of glutamatergic neurons. This results in a transient increase in glutamate release and preferential activation of postsynaptic AMPA (α-amino-3-hydroxy-5-methyl-4-isoxazolepropionic acid) receptors, which is considered a key initiating event in the cascade of downstream mechanisms responsible for sustained neuroplastic effects. AMPAR activation induces the release of BDNF and stimulation of the TrkB receptor, thereby triggering parallel intracellular signaling pathways. A central role is played by activation of the PI3K-AKT-mTOR axis, leading to phosphorylation of the mTORC1 complex and enhanced translation of synaptic proteins involved in synaptogenesis and stabilization of synaptic connections. In parallel, inhibition of eukaryotic elongation factor 2 kinase (eEF2K) results in deactivation of eEF2, relieving translational repression of BDNF synthesis and further amplifying neurotrophic signaling. Concurrent activation of the ERK/MAPK pathway promotes the expression and translation of key structural and functional synaptic proteins, including the AMPA receptor subunit GluR1, the postsynaptic density protein PSD-95, and synapsin I. BDNF-dependent signaling further increases the expression and synaptic incorporation of Ca^2+^-permeable AMPA receptors (CP-AMPARs), primarily in the form of GluR1 homomers. This process is additionally modulated by activation of CaMKK through a TRPC channel–dependent mechanism, which enhances AKT activation and facilitates stable integration of CP-AMPARs into the postsynaptic membrane. The resulting increase in Ca^2+^ influx promotes further strengthening of synaptic plasticity signaling and long-term potentiation processes. Collectively, these precisely coordinated downstream mechanisms lead to rapid reorganization of synaptic architecture and sustained enhancement of neuronal connectivity, forming the molecular basis of ketamine’s enduring antidepressant effects [42,43].

The first clinical evidence for the antidepressant properties of ketamine was reported by Berman et al., who demonstrated that a single intravenous administration of a subanesthetic dose of ketamine (0.5 mg/kg) produced a significant antidepressant effect within a few hours [44]. A major clinical milestone was achieved in March 2019, when the FDA approved intranasal esketamine as an adjunctive treatment to standard pharmacotherapy in patients with TRD, followed by a similar decision by the European Medicines Agency. In August 2020, the FDA further expanded the indication to include patients with MDD accompanied by suicidal ideation, highlighting the clinical relevance of the rapid onset of action of this therapy [45,46].

From early randomized trials onward, ketamine and esketamine demonstrated significant efficacy in treatment-resistant populations. In a 2012 study, response rates exceeded 50%, and remission was achieved in 30–50% of patients, representing outcomes clearly superior to those observed with classical antidepressant drugs [47]. It should be noted, however, that most early studies were characterized by limited follow-up durations.

The durability of therapeutic effects remains an important clinical issue. In a randomized study, only 25% of patients who continued esketamine treatment experienced relapse within one year, compared with 57% of patients after discontinuation, indicating the potential of maintenance treatment strategies, although further confirmation in long-term studies is required [48].

The global efficacy of ketamine was further supported by a 2022 meta-analysis including 79 studies and 2665 patients, which indicated its usefulness as a medium- and long-term strategy in the treatment of TRD. The authors emphasized, however, substantial heterogeneity of the data and a limited number of studies with extended follow-up periods [49]. Among more recent reports, a randomized phase II trial of an oral extended-release formulation of ketamine demonstrated dose-dependent clinical improvement with good tolerability, while also drawing attention to a pronounced placebo effect [50]. In addition, a secondary analysis of the ELEKT-D trial (2024) showed greater symptom improvement in patients treated with intravenous ketamine compared with electroconvulsive therapy, although interpretation of these findings requires caution due to the limited observation period [51].

Despite accumulating evidence supporting the efficacy of ketamine and esketamine in TRD, important challenges related to safety and unresolved clinical issues remain. Data on long-term use are still limited, particularly with regard to potential risks of neurotoxicity, cognitive impairment, and adverse effects on the urinary system with chronic exposure. Moreover, the dissociative and euphoric properties of ketamine raise concerns about misuse and the development of dependence, justifying the need for strict clinical supervision. Questions regarding optimal dosing, administration frequency, duration of maintenance treatment, and the identification of biomarkers predictive of therapeutic response also remain unresolved. These limitations clearly indicate the need for further long-term randomized studies assessing both the efficacy and safety of ketamine in routine clinical practice [42,43,44,45,46,47,48,49,50,51].

Among the numerous ongoing clinical trials, a search of the ClinicalTrials.gov database identified 374 studies involving ketamine (as of 16 November 2025). Selected studies were used to illustrate the overall direction of current research efforts on this compound and are summarized in Table 2.

#### 2.2.2. Psilocybin

The mechanism of action of psilocybin in the treatment of MDD is based on complex receptor interactions, activation of specific signaling pathways, and cascades of downstream effects leading to persistent neuroplastic and functional changes within neuronal circuits regulating mood. After dephosphorylation to its active form—psilocin—this compound acts primarily as a potent agonist of serotonin 5-HT_2_A receptors, which are abundantly expressed in the neocortex, particularly in the prefrontal cortex. 5-HT_2_A agonism initiates a characteristic, selectively “biased” receptor signaling profile typical of classical psychedelics, involving simultaneous activation of Gq/11 and Gi/o proteins through a mechanism dependent on the Gβγ subunit and Src kinase, as well as functional coupling of the 5-HT_2_A receptor with the metabotropic glutamate receptor type 2 (mGlu_2_) in the form of a 5-HT_2_A/mGlu_2_ heteroreceptor complex. Activation of this complex triggers signaling cascades distinct from those induced by non-hallucinogenic 5-HT_2_A agonists, leading to selective induction of immediate early genes such as egr-1 and egr-2, which are directly involved in the regulation of synaptic plasticity.

In parallel, 5-HT_2_A agonism modulates glutamatergic transmission by increasing the excitability of prefrontal cortical pyramidal neurons and enhancing glutamate release, thereby promoting activation of AMPA and NMDA receptors. At the downstream level, this leads to increased expression of BDNF and activation of the BDNF-TrkB axis, which initiates the PI3K-AKT, Ras/MAPK, and PLCγ-Ca^2+^ signaling cascades. These pathways converge on transcription factors, including CREB, thereby promoting synaptogenesis, spinogenesis, and long-term synaptic potentiation processes. In addition, psilocybin may induce secondary downregulation of 5-HT_2_A receptor expression, which may contribute to the persistence of antidepressant and anxiolytic effects following single or limited exposure. A complementary downstream mechanism involves immunomodulatory effects, consisting of inhibition of proinflammatory cytokine release, such as TNF-α and IL-6, via 5-HT_2_A agonism, potentially attenuating the inflammatory component of MDD. Collectively, these mechanisms lead to transient destabilization of pathological patterns of neuronal network activity and to sustained improvement in neuroplasticity and functional connectivity, constituting the biological basis of the antidepressant effects of psilocybin [52].

Interest in the clinical potential of psilocybin was preceded by preclinical studies demonstrating that this compound rapidly reverses anhedonia-like behaviors in animal models and enhances synaptic strength in the hippocampus, exhibiting effects comparable to those of classical antidepressant drugs [53]. These findings provided the foundation for subsequent translational studies in humans.

In a randomized phase II clinical trial involving adult patients with TRD, participants were randomly assigned to receive a single dose of a patented synthetic formulation of psilocybin (25 mg, 10 mg, or 1 mg), administered in combination with standardized psychological support. It was demonstrated that the 25 mg dose—unlike the 10 mg dose—resulted in a clinically significant reduction in depressive symptom severity over a three-week period compared with the 1 mg dose, confirming a dose-dependent therapeutic effect [54]. In another study involving patients with TRD, psilocybin administration led to clinical improvement in all participants within the first week after dosing, with nearly half of the patients maintaining a therapeutic response for up to five weeks [55].

Neuroimaging studies have also provided important insights. Functional magnetic resonance imaging (fMRI) analyses revealed reduced cerebral blood flow within the amygdala, correlated with mood improvement, as well as increased functional connectivity within the default mode network (DMN) following psilocybin treatment. These findings suggest that the antidepressant effects may be partially mediated by functional reorganization of neuronal networks and attenuation of rigid, pathological patterns of brain activity [55]. These observations were complemented by qualitative analyses of patient experiences, which showed that psilocybin can induce intense emotional experiences, encompassing both positive states and subjectively challenging affective phenomena [56].

Despite promising preclinical and clinical findings, the use of psilocybin in the treatment of MDD is associated with significant limitations and unresolved safety concerns. Available clinical data are derived mainly from studies with small sample sizes, short follow-up periods, and intensive psychological support, which complicates unequivocal assessment of the independent pharmacological effect. Considerable interindividual variability in therapeutic response and the induction of highly intense emotional experiences may lead to subjective discomfort or psychological destabilization in some patients. The most commonly observed adverse effects include nausea, headache, dizziness, transient perceptual disturbances, and anxiety occurring during or immediately after the therapeutic session. Although these symptoms are generally short-lived and self-limiting, their occurrence underscores the need for close patient monitoring and appropriate psychological preparation prior to intervention. Moreover, the long-term consequences of repeated exposure to 5-HT_2_A receptor agonism—including the potential risk of persistent perceptual disturbances or other adverse neuropsychiatric changes—remain insufficiently characterized. Determination of optimal dosing regimens, patient selection criteria, and standardization of therapeutic protocols also remains a major challenge, clearly indicating the need for further large-scale, long-term randomized studies assessing both the efficacy and safety of psilocybin in the treatment of MDD [52,53,54,55,56].

According to the ClinicalTrials.gov registry, as of 16 November 2025, 92 ongoing or completed clinical trials involving psilocybin have been identified. Selected representative studies are summarized in Table 3 to illustrate current directions in the development of this therapeutic strategy.

#### 2.2.3. Dextromethorphan–Bupropion

The mechanism of action of the dextromethorphan-bupropion combination in the treatment of MDD involves complex receptor interactions, modulation of key signaling pathways, and cascades of downstream effects that influence the functioning of neuronal networks regulating mood. Dextromethorphan acts as a non-competitive antagonist of N-methyl-D-aspartate receptors (NMDARs), thereby modulating excessive glutamatergic transmission and indirectly promoting enhanced AMPA receptor-dependent signaling. Concurrently, dextromethorphan exhibits agonism at the sigma-1 receptor, initiating intracellular neuroprotective and pro-plastic mechanisms, including regulation of calcium homeostasis and activation of the PI3K-AKT and MAPK/ERK pathways. At the downstream level, increased BDNF expression and activation of the BDNF-TrkB axis are observed, leading to engagement of the PI3K-AKT-mTOR and Ras/MAPK cascades. These processes result in enhanced synaptogenesis, increased number and stability of dendritic spines, and strengthening of long-term synaptic plasticity within the prefrontal cortex and limbic structures. Bupropion, through potent inhibition of the CYP2D6 isoenzyme, increases the bioavailability of dextromethorphan and additionally modulates noradrenergic and dopaminergic neurotransmission, which may further potentiate downstream effects related to improvements in motivation, affect regulation, and cognitive function. Collectively, these mechanisms promote normalization of pathological patterns of neuronal network activity and sustained reduction in depressive symptoms in patients with MDD [57,58].

The clinical efficacy of the dextromethorphan–bupropion combination has been demonstrated in a randomized, double-blind phase II clinical trial, which showed significantly greater antidepressant efficacy of AXS-05 compared with bupropion monotherapy in patients with moderate to severe MDD. Combination treatment resulted in a more rapid and more pronounced reduction in depressive symptoms. The primary endpoint, defined as the change in total score on the Montgomery-Åsberg Depression Rating Scale (MADRS), was met with high statistical significance and a large effect size. The mean between-group difference was approximately 5 points on the MADRS in favor of AXS-05, representing a clinically meaningful improvement and exceeding the typical differences observed in trials comparing antidepressants with placebo. The superiority of the combination therapy was evident as early as two weeks after treatment initiation and persisted until the end of the study, accompanied by higher rates of remission and clinical response, as well as consistent improvements in global assessments and patient-reported outcomes [59].

In the randomized, double-blind phase III GEMINI trial, AXS-05 was shown to be significantly more effective than placebo in the treatment of moderate to severe unipolar depression. Combination therapy led to rapid and sustained reductions in depressive symptoms, with a statistically significant advantage over placebo observed as early as the first week of treatment. The primary endpoint-change in total MADRS score after six weeks of therapy—was achieved with a clinically meaningful difference in favor of AXS-05. The effect size increased over time and exceeded values typically reported in registration trials of antidepressant drugs. The clinical relevance of these findings was further supported by higher rates of remission and therapeutic response, as well as consistent improvements across multiple scales assessing symptoms, functioning, and quality of life [60].

Despite promising efficacy results, the safety profile of the dextromethorphan-bupropion combination requires cautious interpretation due to limitations of the available evidence base and tolerability signals observed already in early-phase studies. In the six-week phase II trial, the incidence of any adverse event was high in both study arms (72.9% vs. 64.6%), and although most events were mild to moderate in severity, serious adverse events were also reported (6.3% vs. 2.1%), along with identical rates of treatment discontinuation due to adverse events (12.5% in both groups). Of particular note was the higher incidence of dizziness and anxiety in the AXS-05 group (e.g., dizziness: 20.8% vs. 4.2%), which in some cases led to treatment discontinuation and may have implications for adherence in real-world clinical settings. At the same time, the absence of severe adverse events and the lack of a signal for increased suicidal behavior during the short observation period do not resolve questions regarding long-term safety, rare adverse events, or risks in patient populations typically excluded from clinical trials. Controversy also surrounds the interpretation of the absence of psychotomimetic signals-although AXS-05 did not exhibit such a profile in clinical studies, the observation of a single psychotic episode in the control arm underscores the need for continued long-term monitoring of neuropsychiatric symptoms, particularly in the context of glutamatergic modulation [57,58,59,60].

According to data from the ClinicalTrials.gov registry, nine clinical trials are currently registered evaluating the use of the dextromethorphan–bupropion combination in the treatment of depression. A summary of these studies, together with brief characteristics, is presented in Table 4.

#### 2.2.4. Zuranolone

In response to the specific pathophysiological challenges associated with postpartum depression, zuranolone—a novel, orally administered modulator of γ-aminobutyric acid type A (GABA-A) receptors—has been developed. Zuranolone belongs to the class of selective allosteric GABA-A modulators and was designed to provide a rapid onset of action. It has been approved by FDA for the treatment of postpartum depression, representing the first oral therapy directly targeting GABAergic inhibitory dysfunction in this condition [61]. Its mechanism of action differs substantially from both classical antidepressants that modulate monoaminergic neurotransmission and benzodiazepines, resulting in a distinct pharmacodynamic profile and potentially different neurobiological consequences.

Zuranolone binds allosterically to the GABA-A receptor at the interface between the α and β subunits, a site widely represented across most receptor isoforms, including both synaptic and extrasynaptic GABA-A receptors. In contrast to benzodiazepines, which primarily interact with the α-γ subunit interface of synaptic GABA-A receptors and predominantly enhance fast, phasic inhibitory currents, zuranolone potentiates both synaptic (phasic) and extrasynaptic (tonic) inhibition. Tonic GABAergic currents, generated by high-affinity extrasynaptic GABA-A receptors, play a crucial role in regulating baseline neuronal excitability and stabilizing neuronal network activity. At the cellular level, activation of GABA-A receptors by zuranolone leads to increased chloride ion influx, membrane hyperpolarization, and a reduced probability of action potential generation. This effect initiates downstream cascades involving normalization of dysregulated calcium-dependent signaling pathways, reduction in excessive glutamatergic neuronal activity, and secondary modulation of gene expression related to synaptic plasticity and stress responses. In vitro data further suggest that, unlike benzodiazepines—which may induce GABA-A receptor internalization and reduce surface receptor density—zuranolone promotes increased surface expression of GABA-A receptors, potentially supporting sustained efficacy of GABAergic inhibition over time. Collectively, by simultaneously enhancing phasic and tonic inhibition and stabilizing GABA-A receptor availability, zuranolone may rapidly restore excitatory-inhibitory balance within neuronal circuits disrupted in postpartum depression. Normalization of this balance enables more adaptive processing of internal and external stimuli and likely constitutes the neurobiological basis of the drug’s rapid clinical onset of action, distinguishing it from traditional antidepressant therapies [62].

Evidence for the clinical efficacy of zuranolone is provided primarily by a randomized phase III clinical trial published in 2023, which included 196 patients with postpartum depression. This study demonstrated a statistically and clinically significant reduction in depressive symptom severity in the zuranolone-treated group compared with placebo, with therapeutic effects observed within 15 days of treatment initiation. Patients receiving zuranolone also achieved significantly better outcomes in global clinical assessments, confirming the clinical relevance of the observed improvement [63].

These findings are supported by a systematic review and meta-analysis published in 2024, which included eight studies assessed both quantitatively and qualitatively. This analysis confirmed the significant efficacy of zuranolone, demonstrating a marked improvement in Hamilton Depression Rating Scale scores compared with placebo, while maintaining a favorable tolerability profile [64].

Despite promising efficacy outcomes and an acceptable safety profile observed in available clinical trials, the use of zuranolone is subject to important limitations related to the nature of the existing evidence. Although no serious adverse events were reported in the phase III trial and the most commonly observed side effects—such as somnolence and dizziness—were mild to moderate in severity [63], these symptoms may have particular clinical relevance in postpartum populations. Fatigue, impaired concentration, and the demands of intensive newborn care may amplify the functional impact of even moderate sedation. Furthermore, although the meta-analysis did not demonstrate an increased overall risk of adverse events compared with placebo [64], the short-term duration of most studies limits assessment of long-term safety, the risk of cumulative sedative effects, and potential interactions with other centrally acting medications. Safety considerations also remain unresolved in broader clinical populations, including patients with comorbid conditions or elevated psychiatric risk. These limitations clearly indicate the need for further post-marketing studies and long-term observational data to better characterize the safety and real-world use of zuranolone in clinical practice.

According to the ClinicalTrials.gov registry, 12 clinical trials are currently registered evaluating the use of zuranolone in the treatment of depression. Selected key studies are summarized in Table 5 to illustrate current directions in the development of this therapeutic strategy.

#### 2.2.5. Brexanolone

Brexanolone is a neuroactive steroid that acts as a positive allosteric modulator of GABA-A receptors. Although its precise antidepressant mechanism has not been fully elucidated, available evidence clearly indicates a central role of GABAergic signaling modulation in its therapeutic effects. Brexanolone is chemically identical to allopregnanolone—an endogenous metabolite of progesterone—which underlies its pharmacodynamic properties and provides a rationale for its use in mood disorders associated with abrupt hormonal fluctuations [65]. In 2019, brexanolone was approved by FDA for the treatment of postpartum depression, and its clinical development and therapeutic indication are conceptually aligned with those of the subsequently approved zuranolone [66].

At the receptor level, brexanolone binds allosterically to GABA-A receptors, which are pentameric chloride-permeable ion channels and constitute the principal inhibitory mechanism in the central nervous system. This modulation enhances the receptor’s response to endogenous GABA, resulting in increased Cl^−^ influx into neurons, membrane hyperpolarization, and reduced neuronal excitability. In contrast to benzodiazepines, which primarily target synaptic GABA-A receptors via allosteric sites located at the α-γ subunit interface, neurosteroids—including brexanolone—bind to distinct allosteric sites, enabling modulation of both synaptic and extrasynaptic GABA-A receptors. Consequently, brexanolone potentiates not only fast, phasic GABAergic inhibition but also tonic inhibition, which is critical for regulating baseline neuronal excitability and stabilizing neuronal network activity.

At the downstream level, enhancement of GABAergic signaling leads to normalization of excessive activity within cortico-limbic circuits involved in mood regulation, anxiety responses, and stress processing. Neuronal hyperpolarization secondarily limits pathological Ca^2+^ influx, thereby influencing calcium-dependent intracellular signaling pathways, including protein kinase cascades and transcription factors involved in synaptic plasticity and adaptive stress responses. These mechanisms likely underlie the rapid improvement in depressive symptoms observed clinically following brexanolone administration. The particular therapeutic relevance of brexanolone in postpartum depression is linked to its ability to compensate for abrupt neurosteroid fluctuations occurring after childbirth. The physiological increase in allopregnanolone levels during pregnancy, followed by a sharp decline in the postpartum period, is considered a key pathogenetic factor in postpartum depression. By mimicking the effects of endogenous allopregnanolone, brexanolone restores appropriate modulation of GABA-A receptors and stabilizes inhibitory neuronal network activity. Additionally, it has been suggested that brexanolone may promote functional normalization of dysregulated GABA-A receptors, abnormalities of which have been described not only in postpartum depression but also in anxiety disorders and other neuropsychiatric conditions [67].

The clinical efficacy of brexanolone has been confirmed in analyses encompassing both phase II and phase III trials. A post hoc analysis published in 2021, which included a total of three phase II and two phase III studies involving 209 patients with postpartum depression, demonstrated a statistically significant and clinically meaningful improvement in mental health status among women treated with brexanolone compared with placebo. A characteristic feature of the clinical response was the rapid onset of action—observed within the first 60 h of treatment initiation—and the persistence of therapeutic effects for up to 30 days after treatment completion, clearly distinguishing brexanolone from classical antidepressant drugs [68].

Consistent results were obtained in a pooled analysis of data from three pivotal randomized phase II and III clinical trials involving 209 women with moderate to severe postpartum depression. Brexanolone was shown to provide a rapid onset of action, with significant improvements in depressive symptoms, anxiety, and sleep disturbances observed within 24–60 h of initiation of the 60 h intravenous infusion. Clinical response, assessed using the Hamilton Depression Rating Scale (HAMD-17) and the CGI-I scale, was significantly more frequent and faster compared with placebo and was maintained for up to 30 days after treatment completion. Improvements in anxiety and sleep are of particular clinical relevance given their frequent co-occurrence in postpartum depression and their negative impact on disease course [69].

Despite a well-characterized adverse effect profile, the safety and scope of potential brexanolone use remain subjects of ongoing evaluation. The most commonly observed adverse effects include somnolence, sedation, altered mental status, and episodes of loss of consciousness, which—although predictable in the context of enhanced GABA-A receptor modulation—represent important clinical limitations and necessitate close patient monitoring as well as avoidance of activities requiring full psychomotor alertness during treatment [67]. Additional concerns relate to the potential accumulation of the sodium salt of sulfobutyl ether β-cyclodextrin, used as a drug carrier, in patients with renal impairment, which may limit the safety of therapy in this population. Importantly, despite the well-documented efficacy of brexanolone in postpartum depression, available data on its safety and effectiveness in the treatment of MDD remain very limited. This underscores the need for further well-designed clinical studies assessing both short- and long-term safety of brexanolone in broader populations of patients with depression before consideration of expanding its therapeutic indications [65,66,67,68,69].

According to the ClinicalTrials.gov registry, 11 clinical trials are currently registered evaluating the use of brexanolone in the treatment of depression. Selected key studies are summarized in Table 6.

#### 2.2.6. Gepirone ER

Gepirone exerts its antidepressant effects through selective modulation of the serotonergic system, with a particular emphasis on 5-HT1A receptors, which clearly distinguishes it mechanistically from classical antidepressants based on non-selective monoaminergic modulation. As a 5-HT1A receptor agonist, it acts via a dual mechanism: presynaptically on autoreceptors located in neurons of the raphe nuclei, and postsynaptically within key limbic and cortical structures involved in the regulation of mood, anxiety, and cognitive function, including the hippocampus, prefrontal cortex, and amygdala. During the initial phase of treatment, activation of presynaptic 5-HT1A autoreceptors leads to transient inhibition of serotonergic neuronal firing and reduced serotonin release; however, with chronic administration, these autoreceptors undergo desensitization, internalization, and reduced coupling to Gi/o proteins. As a consequence of these neuroadaptive changes, a secondary and sustained increase in serotonin release occurs in projection areas of the brain, constituting a key component of the long-term antidepressant effect.

At the postsynaptic level, activation of 5-HT1A receptors by gepirone initiates G protein–dependent signaling cascades, leading to modulation of intracellular pathways, including the cAMP/PKA, PI3K/AKT, and MAPK/ERK axes. Activation of these pathways influences ion channel conductance, synaptic plasticity, and gene expression related to neuroadaptation, neurogenesis, and neuronal survival. Downstream effects include improved functioning of neuronal networks responsible for emotional and stress processing, translating into a reduction in depressive and anxiety symptoms. In addition, active metabolites of gepirone-particularly 3′-hydroxygepirone, a 5-HT1A agonist, and 1-(2-pyrimidinyl)piperazine (1-PP), which acts as an antagonist of presynaptic α2-adrenergic receptors- may synergistically enhance monoaminergic neurotransmission, further potentiating the therapeutic effect. Collectively, the mechanism of action of gepirone involves precise receptor-level modulation and long-term intracellular signaling changes, leading to sustained improvement in neuronal function disrupted in major depressive disorder [70,71,72].

The clinical efficacy of gepirone has been evaluated in a series of controlled trials and summarized in a systematic review with meta-analysis, which provided meaningful evidence supporting its therapeutic potential in the treatment of MDD. Of the seven controlled studies analyzed, four—after inclusion of pilot analyses—demonstrated consistent and clinically relevant efficacy of gepirone in reducing the severity of depressive symptoms, allowing the authors to conclude that this compound possesses genuine therapeutic value [73]. The most compelling data was reported by Phillips [74], who showed that in the pivotal phase III trials (134001 and FKGBE007), gepirone produced statistically and clinically significant improvements in depressive symptoms compared with placebo, while maintaining a favorable tolerability profile. Reported adverse events, such as dizziness and nausea, were most commonly mild to moderate in severity. Although not all trials unequivocally confirmed gepirone’s efficacy—including studies 134002, FKGBE008, and 134023—these findings should be interpreted in the context of heterogeneity in study design, differences in clinical populations, and variability in selected endpoints.

An additional clinical advantage of gepirone is its favorable impact on sexual function—one of the key factors limiting adherence to antidepressant treatment. Data from five randomized phase III trials demonstrated that extended-release gepirone (ER) significantly outperformed SSRIs in preserving sexual function across all assessed time points [75]. In light of these findings, gepirone ER received FDA approval for the treatment of MDD in the United States in September 2023, confirming its clinical efficacy and representing an important step toward more precise and better-tolerated antidepressant therapies [76].

Despite confirmed clinical efficacy, the full long-term safety profile of gepirone ER remains incompletely characterized. Available data indicate that the drug is generally well tolerated, with the most commonly reported adverse effects—such as dizziness, nausea, headache, and somnolence—being typically mild to moderate in severity and rarely leading to treatment discontinuation. The absence of clinically significant sexual dysfunction represents a clear advantage over SSRIs. At the same time, an important limitation of the current evidence base is the lack of ongoing clinical trials evaluating gepirone in MDD, suggesting a potential knowledge gap regarding long-term safety, use in specific clinical populations, and drug–drug interactions. Therefore, despite its promising efficacy and tolerability profile, continued post-marketing surveillance and observational studies remain necessary to precisely define the role of gepirone in long-term treatment strategies for depression [70,71,72,73,74,75,76].

#### 2.2.7. Cariprazine

Cariprazine exerts antidepressant effects through precise modulation of dopaminergic and serotonergic neurotransmission, acting as a partial agonist at dopamine D3 and D2 receptors—with a marked preferential affinity for D3 receptors—and as a partial agonist at 5-HT1A receptors [77]. It’s very high affinity for D3 receptors is of particular therapeutic relevance, as these receptors are predominantly localized within mesolimbic and mesocortical circuits responsible for reward processing, motivation, psychomotor drive, and the anhedonic component of depression. Through partial agonism, cariprazine stabilizes dopaminergic signaling by enhancing transmission under conditions of dopaminergic deficit characteristic of MDD, while simultaneously preventing excessive receptor activation.

At the molecular level, activation of Gi/o protein–coupled D3/D2 receptors leads to inhibition of adenylyl cyclase and a reduction in cyclic adenosine monophosphate (cAMP) levels, thereby initiating signaling cascades involving modulation of protein kinases PKA, PI3K/AKT, and MAPK/ERK. The downstream effects of these pathways include regulation of gene expression associated with synaptic plasticity, dendritic spine remodeling, mitochondrial function, and neuroprotective processes. These changes promote long-term normalization of mesolimbic and cortico-limbic circuit activity, translating into improvements in motivational, emotional, and psychomotor functions. In addition, partial agonism at 5-HT1A receptors may secondarily modulate dopamine release within reward-related regions and activate serotonin-dependent neuroplasticity pathways, further enhancing antidepressant effects. Collectively, downstream effects include improved encoding of reward value, increased reward responsiveness, reduction in anhedonia, and normalization of psychomotor speed. Moreover, it has been suggested that cariprazine’s influence on inflammatory and metabolic processes linked to dopaminergic dysfunction may further support restoration of neuronal homeostasis. As a result, cariprazine targets key pathophysiological domains of depression—particularly motivational deficits and anhedonia—providing a biological basis for its clinical efficacy [78,79,80].

The clinical efficacy of cariprazine in the treatment of MDD has been demonstrated in phase III clinical trials evaluating its use as adjunctive therapy in adult patients with an inadequate response to standard antidepressant treatment. In a randomized, double-blind, placebo-controlled study, doses of 2–4.5 mg/day were significantly superior to placebo in reducing depressive symptom severity as measured by the MADRS after 8 weeks of treatment (LSMD—2.2 points), with onset of effect observed as early as the second week of therapy. Treatment was also associated with higher rates of clinical response assessed using the MADRS and CGI-I scales. The lower dose range (1–2 mg/day) did not achieve statistical significance for the primary endpoint, although it increased response rates. Cariprazine also improved global functioning and illness severity as assessed by CGI scales; however, its impact on social and occupational functioning was limited [78].

The consistency and robustness of the therapeutic effect were confirmed by a systematic review and meta-analysis encompassing five randomized, double-blind clinical trials involving a total of 2013 adult patients with MDD or TRD treated with cariprazine at doses of 1–4.5 mg/day for 6–26 weeks. Adjunctive cariprazine was associated with a statistically significant and clinically consistent reduction in depressive symptom severity compared with placebo. This effect was consistently observed across key depression rating scales, including MADRS (LSMD −1.88), CGI-S (LSMD −0.18), and HAMD-17 (LSMD −0.96), with no significant heterogeneity between studies (I^2^ = 0%), indicating high reproducibility of the therapeutic effect regardless of differences in dosing and treatment duration [81].

Notably, cariprazine also demonstrates considerable clinical versatility. It is approved for the treatment of schizophrenia, acute manic and mixed episodes, and bipolar depression, underscoring its broad capacity to modulate pathological dopaminergic and affective states [82].

The safety profile of cariprazine used as adjunctive therapy in MDD indicates predictable but clinically relevant limitations typical of atypical antipsychotics, particularly with respect to activation-related symptoms and extrapyramidal side effects. Meta-analytic data revealed a significantly increased risk of psychomotor agitation, akathisia, tremor, and somnolence, as well as more frequent occurrence of fatigue and nausea. Akathisia showed substantial variability across studies, suggesting potential dependence on dose, duration of exposure, and characteristics of the clinical population. Although most adverse events were mild to moderate in severity, their nature may substantially affect treatment tolerability, adherence, and continuation decisions. At the same time, the absence of a strong signal for significant weight gain and certain metabolic adverse effects represents a potential advantage of cariprazine over other augmentation strategies. Nevertheless, short observation periods and heterogeneity in dosing regimens limit conclusions regarding long-term safety. Consequently, despite confirmed clinical efficacy, the use of cariprazine in MDD remains associated with unresolved issues concerning optimal dosing, long-term tolerability, and identification of patients at increased risk for adverse effects, justifying the need for further well-designed studies [78,81,82].

According to the ClinicalTrials.gov registry, 18 clinical trials are currently registered evaluating the use of cariprazine in the treatment of depression. Selected key studies are summarized in Table 7.

#### 2.2.8. (R)-Ketamine

(R)-Ketamine is the enantiomer of ketamine and exhibits antidepressant effects that do not directly correlate with its affinity for the NMDA receptor, indicating that classical NMDAR inhibition does not constitute the dominant mechanism of its action. Although (R)-ketamine may indirectly influence glutamatergic neurotransmission, available evidence suggests that its sustained therapeutic effects do not arise from persistent NMDAR blockade but rather from the engagement of secondary signaling mechanisms. Activation of AMPA receptors is essential for initiation of the antidepressant response, leading to increased activity of glutamatergic neurons and triggering intracellular signaling cascades.

A central component of the mechanism of (R)-ketamine is activation of the BDNF-TrkB pathway. (R)-Ketamine induces an increase in BDNF levels and activation of its receptor TrkB in the prefrontal cortex and hippocampus, resulting in engagement of the ERK signaling cascade. ERK activation leads to phosphorylation of the transcription factor CREB and enhanced expression of genes involved in neuroplasticity, neuronal survival, and stabilization of synaptic connections. An important complementary mechanism involves microglia-dependent TGF-β1 signaling. (R)-Ketamine enhances TGF-β1 signaling, which is required for the manifestation of its antidepressant effects, indicating a contribution of neuro-immune interactions to its mechanism of action. Activation of this pathway cooperates with neurotrophic mechanisms, promoting long-lasting structural and functional changes within limbic–cortical circuits.

**Figure 2:**
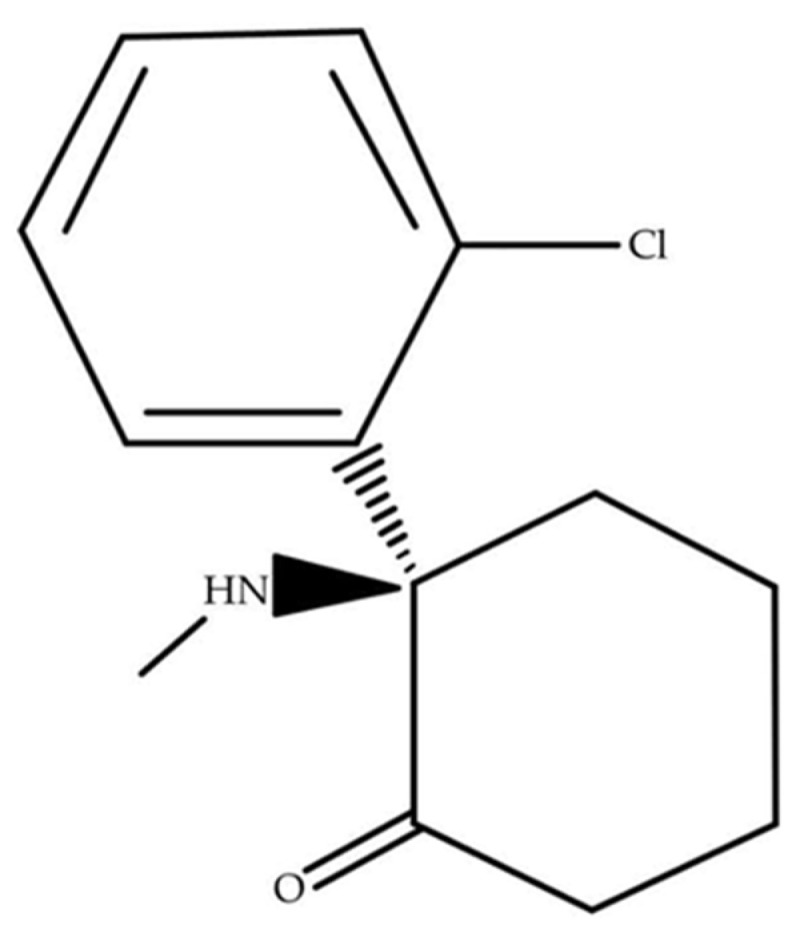
Structural formula of (R)-ketamine.

Downstream, (R)-ketamine leads to normalization of synaptic plasticity, including increased dendritic spine density and restoration of appropriate expression of postsynaptic proteins in the prefrontal cortex and hippocampus. These effects are sustained and correlate with persistent improvement in depression-like behaviors in stress-based models, indicating that the principal outcome of (R)-ketamine treatment is long-term functional remodeling of neuronal networks rather than transient modulation of a single receptor target [83,84,85]. The chemical structure of this compound is presented as a structural formula in Figure 2.

A 2021 rat study demonstrated that the sustained antidepressant effects of (R)-ketamine in a chronic social defeat stress model are mediated by activation of the microglial ERK-NRBP1-CREB-BDNF pathway in the medial prefrontal cortex (mPFC). iTRAQ-based proteomic analysis identified NRBP1 as a protein differentiating the response to (R)-versus (S)-ketamine; NRBP1 is expressed in microglia and functionally interacts with the transcription factor CREB. Compared with (S)-ketamine, (R)-ketamine more robustly induced ERK activation, CREB phosphorylation, and increased BDNF expression in microglia, whereas pharmacological inhibition of ERK abolished these effects, indicating its upstream position in this cascade. Activated CREB directly enhanced transcription of Bdnf exon IV, as confirmed by promoter assays and by selective knockdown of CREB and Bdnf exon IV, which eliminated both the molecular and behavioral antidepressant effects of (R)-ketamine. The critical role of microglia was further supported by findings that partial microglial depletion or inhibition of the anti-inflammatory microglial phenotype resulted in loss of (R)-ketamine efficacy. Downstream, activation of the ERK-NRBP1-CREB-BDNF pathway led to normalization of impaired synaptic plasticity in the mPFC, including restoration of dendritic spine density. Collectively, these results indicate that microglial ERK-NRBP1-CREB-BDNF signaling constitutes a key mechanism underlying the sustained antidepressant effects of (R)-ketamine [86].

A 2020 study provided the first clinical evaluation of the antidepressant efficacy of (R)-ketamine in humans and delivered preliminary but clinically meaningful evidence of its therapeutic potential in TRD. A single intravenous infusion of (R)-ketamine (0.5 mg/kg) produced a rapid and pronounced antidepressant effect, observable within 60 min and peaking at 240 min, with improvement persisting for up to 7 days after administration. The magnitude of symptom reduction assessed using the MADRS was substantial, with high response and remission rates, particularly during the first hours following infusion. Importantly from a clinical perspective, (R)-ketamine demonstrated a favorable tolerability profile, with minimal dissociative symptoms and only minor changes in hemodynamic parameters. Although these findings should be interpreted with caution due to the open-label design and small sample size, they provide proof of concept that (R)-ketamine may represent a promising, rapidly acting therapeutic candidate for TRD, justifying further randomized, placebo-controlled trials [87].

Perception neurosciences have invested substantial resources in the clinical development of (R)-ketamine, conducting several clinical studies to evaluate its therapeutic potential. In two phase II trials involving intravenous administration in patients with TRD, antidepressant effects of the investigational compound were observed; however, these studies did not meet their prespecified primary endpoints. Notably, post hoc analyses revealed clinically meaningful reductions in depressive symptom severity persisting for up to two weeks following a single administration of (R)-ketamine, with remission within 24 h reported in 43% of study participants [88].

Despite encouraging preclinical findings and preliminary clinical observations, the safety profile and therapeutic relevance of (R)-ketamine remain insufficiently defined. Current clinical evidence is based on very small sample sizes, which substantially limits reliable assessment of the frequency, nature, and severity of potential adverse events and precludes adequate control for placebo effects. Although available observations did not identify prominent dissociative effects or significant hemodynamic disturbances, systematic data on safety with repeated or long-term administration, potential neurobiological consequences, and abuse liability are lacking. Moreover, translation of molecular and cellular mechanisms identified in animal models-particularly microglia-dependent signaling-into clinical conditions in humans remains uncertain. Consequently, the current state of knowledge supports considering (R)-ketamine as an experimental therapeutic candidate that requires further validation in well-designed, randomized, placebo-controlled studies with sufficiently long follow-up periods [83,84,85,86,87,88].

**Figure 3:**
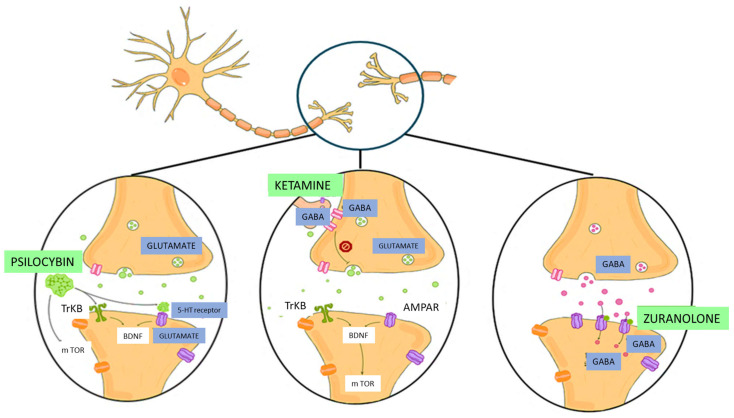
Molecular mechanism of action of ketamine, psilocybin and zuranolone.

Below, Figure 3 presents a graphical comparison of the molecular mechanisms of ketamine, psilocybin, and zuranolone.

Below, Table 8 presents a summary of the mechanisms of action of novel antidepressant drugs, together with their synaptic effects and downstream mechanisms.

#### 2.2.9. TMS—Transcranial Magnetic Stimulation

TMS is a non-invasive technique based on the stimulation of neurons using electromagnetic fields. A coil placed near the scalp generates a magnetic field that penetrates the skull and induces electric currents in the cerebral cortex, thereby modulating neuronal excitability. Repetitive TMS (rTMS) can either increase or decrease cortical activity at the site of stimulation as well as in interconnected brain regions involved in mood regulation and cognitive functions [89].

In the treatment of MDD, rTMS exerts its effects primarily through synaptic mechanisms of neuronal plasticity resembling long-term potentiation (LTP) and long-term depression (LTD), which represent functional correlates of sustained changes in synaptic efficacy. At the level of receptor interactions, a key mechanism involves alterations in synaptic transmission efficiency within stimulated or inhibited cortical regions, leading to durable modulation of neuronal excitability in structures regulating emotion, particularly the left dorsolateral prefrontal cortex (DLPFC). At the intracellular level, the induction of LTP- or LTD-like changes is associated with activation of signaling cascades responsible for consolidating plastic changes that stabilize increases or decreases in synaptic strength. These mechanisms result in long-term regulation of neuronal activity and synchronization of neuronal networks, including modulation of oscillatory dynamics within cortico-subcortical circuits implicated in the pathophysiology of depression. Downstream effects include reconstruction of the functional organization of cortical networks, characterized by restoration of balance between hyperactive and hypoactive brain regions. These changes correlate with clinical improvement of depressive symptoms and are long-lasting, although their magnitude is influenced by individual neuroplastic potential, which declines with age. Overall, rTMS harnesses LTP/LTD-dependent synaptic plasticity as a mechanism enabling adaptive reorganization of neuronal networks conducive to mood improvement [90].

In 2023, a randomized study investigated the mechanisms underlying TMS therapy in individuals with depression. Treatment resulted in a significant increase in activity within the dorsolateral prefrontal cortex, hippocampus, and orbitofrontal cortex. Notably, increased hippocampal activity was strongly correlated with symptom reduction. These findings highlighted the crucial role of the orbitofrontal–hippocampal pathway in the antidepressant effects of TMS [91]. A meta-analysis of 10 studies conducted by Sigrist C. et al. demonstrated that TMS can be an effective treatment for depression in both younger individuals and patients with severe disease. The authors emphasized that therapeutic efficacy may be enhanced through optimization of treatment protocols, such as increasing the number of sessions, extending treatment duration, or employing unilateral stimulation strategies [92].

With respect to late-life depression, a systematic review including seven randomized controlled trials and seven uncontrolled studies (total n = 420) was conducted to update evidence on the efficacy of TMS in this population. The analysis indicated that TMS is safe, well tolerated, and shows highly promising efficacy in the treatment of geriatric depression. However, the authors underscored the need for individualized optimization of therapy, as a subset of patients did not exhibit clear clinical improvement [93].

The literature also describes accelerated TMS (aTMS), which aims to shorten treatment duration and accelerate symptom reduction. Early studies suggest comparable efficacy and safety relative to standard rTMS protocols. Nevertheless, challenges remain regarding the identification of key parameters—such as stimulation frequency, number of daily sessions, total pulse count, and target localization methods—required to achieve optimal therapeutic outcomes [94].

#### 2.2.10. Transcranial Direct Current Stimulation (tDCS)

tDCS is a modern therapeutic approach for major depressive disorder that can be applied at early stages of treatment. It involves the delivery of low-intensity direct current (0.5–2 mA) via electrodes placed on the scalp. This current modulates neuronal excitability—anodal stimulation increases excitability, whereas cathodal stimulation decreases it. With repeated sessions, therapeutic effects accumulate over time. In depression-specific protocols, the anode is most commonly positioned over the left dorsolateral prefrontal cortex (DLPFC) to enhance its functional activity [95].

In the treatment of depression with tDCS, the key mechanism of synaptic modulation involves phenomena resembling LTP and LTD within cortical and subcortical networks regulating mood. Anodal stimulation induces a mild, subthreshold depolarization of neuronal membranes beneath the electrode, facilitating Ca^2+^ influx through voltage-gated channels and NMDA receptors, thereby promoting sustained, LTP-like enhancement of synaptic transmission. Conversely, cathodal stimulation produces relative hyperpolarization and reduced excitability, supporting LTD-like processes and long-lasting weakening of synaptic efficacy. At the molecular level, tDCS modulates the expression of glutamatergic receptors (NMDA, AMPA), activates Ca^2+^-dependent signaling cascades (including CREB), induces early response genes such as zif268 and c-fos, and influences BDNF expression. Collectively, these processes consolidate plastic changes—from post-translational modifications to long-term structural remodeling of synapses.

The dependence of tDCS effects on NMDA receptors and their modulation by serotonergic and dopaminergic systems (e.g., prolongation of LTP-like effects after anodal stimulation with increased 5-HT activity, abolition of effects by NMDA or D2 antagonists) indicates that tDCS functions not only as a direct inducer of LTP/LTD-like changes but also as a neuromodulator of the metaplastic “background” that determines the threshold and direction of subsequent plastic changes. In depression, where this homeostasis is disrupted, repeated tDCS sessions may help restore adaptive plasticity within mood-regulating networks by strengthening functionally beneficial connections (LTP-like) and weakening maladaptive ones (LTD-like). Downstream, consolidated NMDA- and Ca^2+^-dependent plastic changes, together with their modulation by monoaminergic systems, lead to a sustained shift in the synaptic plasticity threshold within these networks, stabilizing adaptive patterns of synaptic strengthening and weakening during subsequent neuronal activity—even after stimulation has ended [96].

A 2025 meta-analysis including 5522 participants from 88 randomized clinical trials evaluated the role of tDCS in the treatment of depression. The analysis provided robust evidence that tDCS is an effective adjunctive therapy when combined with pharmacological treatment. The method was generally well tolerated and associated with minimal adverse effects. The authors emphasized the need to optimize stimulation parameters to enable individualized treatment approaches [97]. Particularly noteworthy was a phase II study published in January 2025 that evaluated fully remote, home-based tDCS for the treatment of major depression. Over a 10-week treatment period, participants demonstrated high therapeutic efficacy—significant improvement in depressive symptoms measured by the Hamilton Depression Rating Scale—alongside good acceptability and safety. These findings are especially important as they highlight the potential for treating depression without requiring patients to leave their homes, which represents a significant barrier for many individuals [98].

#### 2.2.11. Photobiomodulation (PBM)

Photobiomodulation primarily employs red light and near-infrared (NIR) light, which can penetrate tissues deeply enough to affect the brain. Red and NIR light act mainly on mitochondria, enhancing cellular metabolism and cerebral blood flow, while also promoting neurogenesis, synaptogenesis, stem cell activation, and increased BDNF levels. PBM has demonstrated therapeutic potential in models of stroke, traumatic brain injury, neurodegenerative diseases, and neuropsychiatric disorders [99].

**Figure 4:**
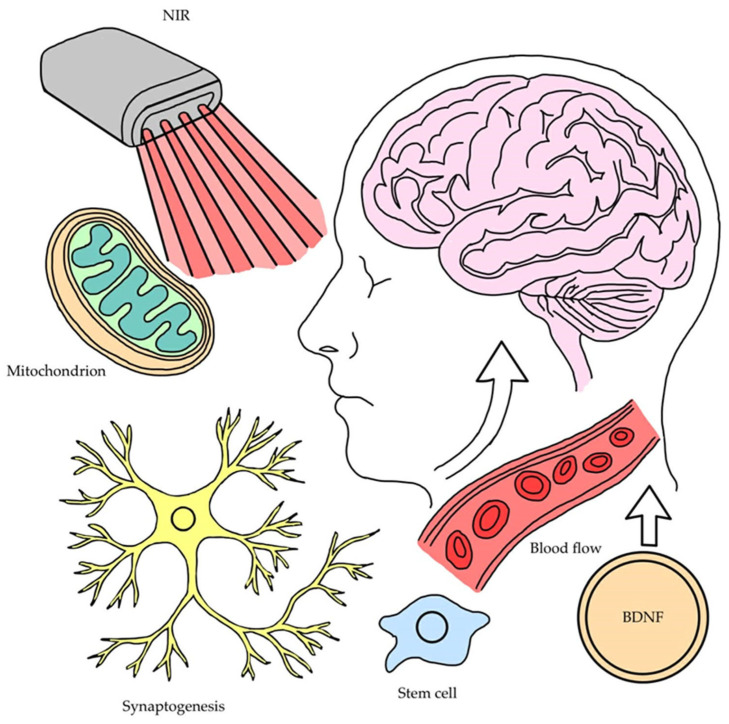
Effects of PBM via NIR light, including mitochondrial modulation, synaptogenesis, increased BDNF, stem cell activation, and improved blood flow.

A schematic illustration of near-infrared light-based photobiomodulation is presented in Figure 4.

As noted above, PBM primarily targets mitochondrial function. Its effects include activation of cytochrome c oxidase, increased activity of respiratory chain enzymes, enhanced ATP production, improved oxygen utilization, and increased cerebral oxygenation. In addition, PBM reduces levels of ROS and pro-inflammatory cytokines. Through this multimodal profile of action, PBM counteracts several key mechanisms implicated in the pathophysiology of depression, thereby conferring substantial therapeutic potential to this intervention [100].

The impact of PBM on depression has been evaluated in a meta-analysis encompassing eleven studies with a total of 407 participants. Data synthesis demonstrated that PBM alleviates depressive symptoms and effectively reduces depression severity. However, due to the relatively limited evidence base, the authors emphasized the need for further high-quality studies to strengthen and extend current findings [101]. To fully harness the therapeutic potential of this modality, the literature consistently highlights the necessity of optimizing stimulation parameters in future research. Such efforts are critical for improving both the efficacy and reproducibility of PBM therapy [102].

## 3. Synthesis and Clinical Implications

The findings of this review confirm that depression—owing to its high prevalence, diverse risk factors, and marked biological heterogeneity—remains one of the major challenges of contemporary medicine. Although classical antidepressants (SSRIs, SNRIs, TCAs, and MAOIs) continue to constitute the backbone of pharmacotherapy, their substantial limitations—including delayed onset of action, adverse effects, and a high rate of treatment-resistant depression—have underscored the need for the development of novel, more precise therapeutic strategies. Contemporary pathophysiological models, emphasizing impaired neuroplasticity, dysregulation of the HPA axis, activation of inflammatory processes, aberrant glutamatergic and GABAergic signaling, and dysfunction of large-scale brain networks, have provided a robust conceptual framework for the development of mechanistically targeted interventions.

The most transformative advances have been observed in therapies modulating glutamatergic neurotransmission. Ketamine and esketamine, owing to their rapid onset of action and well-documented efficacy in TRD, represent a substantial departure from the classical monoaminergic paradigm. Although preliminary data on (R)-ketamine suggest promising therapeutic potential, further clinical studies are required to assess long-term safety and to identify patient populations most likely to benefit from this intervention. An important complement to this approach is the oral combination therapy dextromethorphan–bupropion, which integrates NMDA and sigma-1 receptor modulation with a relatively favorable tolerability profile.

Another significant innovation is represented by GABA-A receptor modulators, which have found particular utility in the treatment of postpartum depression. Both zuranolone and brexanolone enable rapid reduction in depressive symptoms; however, their clinical applicability differs substantially. Oral zuranolone offers the potential for broader clinical use, whereas brexanolone—due to the requirement for intravenous infusion and intensive monitoring—is likely to remain reserved for severe cases. Gepirone ER represents a more selective serotonergic strategy, providing antidepressant efficacy with a reduced risk of sexual dysfunction commonly associated with SSRIs. In contrast, cariprazine, through partial agonism at dopaminergic receptors, appears particularly beneficial in patients with prominent anhedonia, motivational deficits, or treatment-resistant depression.

An important adjunct to pharmacotherapy is provided by non-pharmacological neuromodulation techniques, including TMS, tDCS, and PBM. These interventions align with the modern conceptualization of depression as a disorder of neuronal network plasticity and represent valuable therapeutic options, particularly for patients with insufficient response to pharmacological treatment or with contraindications to its use.

## 4. Materials and Methods

The starting point for preparing this review was a meticulous examination of specialized articles retrieved from the PubMed and Google Scholar databases. In addition, information on clinical trials was obtained from the ClinicalTrials.gov database. To ensure the reliability of the review process, the analysis included clinical studies, review articles, pilot studies, and meta-analyses, provided they addressed depression therapy, with particular emphasis on pharmacological treatment and innovative approaches considered from both holistic and pharmaceutical perspectives. The database search was conducted using the following keywords: antidepressant drug discovery, treatment-resistant depression, neuroinflammation, neuroplasticity, hallucinogens and psychedelics, CNS drug development, molecular targets for depression, neurotransmitter systems. Inclusion criteria encompassed studies with a clear focus on depression and its treatment. Exclusion criteria involved works unrelated to depression or its management, as well as publications whose scientific quality did not meet the required standards. The first step of the analysis consisted of selecting studies that met the inclusion and exclusion criteria. Articles that passed this initial stage were subsequently subjected to detailed scientific evaluation. Particular attention was given to innovative treatment methods, their future development potential, and the possibility of integrating emerging technologies into therapeutic strategies.

During the analysis, several key reference points were identified to structure the work. These included an introductory overview of the established foundations of depression treatment, followed by a comprehensive presentation of newer pharmacological agents that have only recently entered clinical practice or are in the process of doing so. An additional aim was to demonstrate that the management of depression does not have to be limited to pharmacological monotherapy and that interdisciplinary approaches may offer significant benefits. After gathering and examining the relevant information, the data were organized and presented in a logical sequence. Individual sections were arranged coherently to allow for a comprehensive and structured discussion of depression and its treatment. By adhering strictly to the criteria outlined above, it was possible to highlight emerging therapeutic pathways and provide an in-depth analysis of novel strategies in depression management.

## 5. Conclusions

### 5.1. Limitations of Current Pathophysiological Models of Depression

Despite substantial progress in elucidating the biological underpinnings of depression, a single, coherent pathophysiological model that integrates the heterogeneity of clinical symptoms, variability in treatment response, and interactions among genetic, environmental, and neurobiological factors are still lacking. The monoaminergic framework, which dominated for decades, has proven insufficient to explain both the delayed onset of antidepressant efficacy and the high proportion of patients who fail to respond to treatment. Although contemporary concepts incorporate the roles of impaired neuroplasticity, HPA axis dysregulation, inflammatory processes, and mitochondrial dysfunction, these models remain largely fragmentary and are often based on preclinical data whose clinical translatability is limited.

A further challenge lies in the fact that many proposed biomarkers of depression—such as BDNF, inflammatory cytokines, or markers of oxidative stress—exhibit substantial interindividual variability and low diagnostic specificity. This markedly limits their utility in patient stratification and in predicting response to specific therapeutic interventions.

### 5.2. Limitations of Traditional Treatment Mechanisms in Depression

Despite well-documented population-level efficacy, classical antidepressants exhibit several major limitations that undermine their adequacy as universal therapeutic solutions. Most notably, their mechanisms rely on indirect modulation of monoaminergic neurotransmission, resulting in delayed clinical effects that are often inadequate in situations requiring rapid intervention, such as depression accompanied by acute suicidal ideation. Moreover, a substantial proportion of patients experience only partial response or complete treatment resistance, indicating that modulation of serotonin and noradrenaline does not address key pathogenic mechanisms in all individuals.

An additional limitation is the adverse-effect profile, which negatively affects treatment adherence and patients’ quality of life. Sexual dysfunction, weight gain, gastrointestinal symptoms, and sleep disturbances represent significant barriers to long-term use. Furthermore, traditional antidepressants exert limited effects on core depressive domains such as anhedonia, motivational deficits, and cognitive impairment, which are closely linked to dysfunction within dopaminergic and glutamatergic circuits.

### 5.3. Critical Appraisal of Novel Therapeutic Strategies

Despite promising results, novel therapeutic strategies are also subject to important limitations and controversies. Ketamine exemplifies this issue: while its rapid antidepressant effects have transformed conceptual approaches to depression treatment, they have simultaneously revealed substantial methodological challenges. Most studies are characterized by short follow-up periods, and data on long-term safety, optimal dosing regimens, and abuse potential remain limited. Moreover, marked heterogeneity in treatment response suggests that ketamine is not a universal therapy, and the lack of validated response biomarkers complicates its rational clinical implementation.

Similar concerns apply to psychedelic-assisted therapies, including psilocybin. Although clinical trials indicate the potential for sustained improvement following a single intervention, interpretation of these findings is complicated by strong psychotherapeutic context effects, expectancy biases, and inherent difficulties in maintaining blinding. Additional controversy surrounds psychological safety in patients with vulnerability to psychotic or bipolar spectrum disorders.

For GABA-A receptor modulators such as zuranolone and brexanolone, the principal limitations include a narrow range of indications and uncertainty regarding the durability of therapeutic effects after treatment discontinuation. Although rapid onset of action is a clear advantage, the absence of long-term data precludes definitive conclusions about their role in chronic depression management. Dopaminergic strategies, exemplified by cariprazine, are additionally associated with risks of extrapyramidal symptoms and akathisia, which may limit tolerability in certain patient populations.

### 5.4. Inconsistencies and Gaps in the Available Evidence

A major challenge in evaluating novel therapies is the inconsistency of clinical trial results. Differences in study design, inclusion criteria, endpoints, and duration of follow-up hinder direct comparisons across interventions. Gepirone ER illustrates this issue, as several trials failed to demonstrate significant superiority over placebo despite positive findings in registration studies.

Similarly, neuromodulation studies reveal substantial variability in clinical outcomes, depending on stimulation parameters, target localization, and patient characteristics. The lack of standardized protocols and the limited number of comparative trials impede clear identification of optimal therapeutic strategies.

### 5.5. Future Perspectives

The future of depression treatment appears to be moving toward precision-based approaches that integrate clinical, neurobiological, and psychosocial data. A central challenge remains the identification of biomarkers capable of enabling patient stratification and prediction of treatment response. The development of multimodal therapeutic strategies—combining pharmacotherapy, neuromodulation, and psychological interventions—may allow for more effective targeting of the complex pathophysiology of depression.

Another key research direction involves evaluating the long-term effects of emerging therapies, including their impact on neuroplasticity, cognitive functioning, and relapse risk. In this context, pragmatic and observational studies conducted in real-world clinical settings will be of particular importance.

## 6. Summary

Taken together, the innovations discussed in this review underscore a clear shift toward a more precise, individualized, and multimodal approach to the treatment of depression. Despite these promising advances, substantial challenges remain. Data on the long-term safety of many emerging therapies are still limited, comparative effectiveness studies are scarce, and optimal criteria for patient selection have not yet been fully established. Future research should integrate clinical, biological, and digital biomarkers to better inform therapeutic decision-making and to further refine personalized treatment strategies. Overall, the convergence of novel pharmacological agents, neuromodulatory techniques, and digital technologies offers transformative potential to improve treatment outcomes. Continued research, cautious clinical implementation, and broader accessibility will be essential to translate these advances into meaningful and durable improvements in the quality of life of patients suffering from depression.

## Figures and Tables

**Table 1:** Overview of classical antidepressant drugs used in the treatment of depression, with a concise summary of their molecular mechanisms, synaptic effects, and downstream mechanisms.

Drug Class	Primary Molecular Targets/Mechanism	Synaptic Effect	Downstream Mechanisms	Reference
SSRI	Inhibition of the serotonin transporter	Increased serotonin concentration in the synaptic cleft and enhanced activation of postsynaptic 5-HT receptors	- Desensitization of presynaptic 5-HT autoreceptors - Sustained increase in serotonin release - Activation of intracellular cascades regulating gene expression - Enhanced synaptic plasticity - Reduction in negative cognitive biases	[23,24,25,26,27,28]
SNRI	Inhibition of serotonin and norepinephrine transporters	Increased serotonin and norepinephrine concentrations in the synaptic cleft; prolonged neurotransmitter action	- Neuroadaptive changes resulting from chronic monoamine elevation - Increased Cx43 expression in the prefrontal cortex - Normalization of astrocytic gap junctions - Reversal of stress-induced depression-like behaviors	[29,30,31]
TCA	Blockade of presynaptic reuptake of serotonin and norepinephrine	Increased monoaminergic neurotransmission	- Altered expression and sensitivity of monoaminergic receptors - Regulation of intracellular signaling pathways - Antagonism of 5-HT_2_A/5-HT_2_C receptors - Increased cortical release of norepinephrine and dopamine - Increased Cx43 expression and improved astrocytic communication	[32,33,34,35,36,37,38]
MAOI	Irreversible inhibition of monoamine oxidase A and B	Reduced degradation of serotonin, norepinephrine, and dopamine, leading to increased presynaptic and synaptic concentrations	- Long-term regulation of monoaminergic receptors - Activation of neuroplasticity-related signaling pathways - Increased expression of neurotrophic factors - Normalization of neuronal circuits regulating mood	[39,40,41]

**Table 2:** A selection of clinical trials investigating ketamine and its relationship with depression.

Study Name	NCT Number	Brief Description	Study Phase
Ketamine Pharmacokinetics and Pharmacodynamics for Postpartum Depression and Pain After Cesarean Delivery	NCT06767566	The study aims to assess the pharmacokinetics of postpartum ketamine infusion. Its primary objective is to better understand the pharmacokinetics and pharmacodynamics of postpartum ketamine administration. A secondary objective is to compare these parameters with a control group of women of reproductive age and to evaluate differences in ketamine pharmacokinetics in pregnant individuals.	Phase I
The OBSERVE Protocol	NCT06725277	This observational protocol aims to provide long-term follow-up data on patients initiating or continuing treatment with Spravato^®^ or intravenous ketamine. The primary focus is on treatment patterns, long-term effectiveness, and safety outcomes.	Not Applicable
The Improvement of Low-dose Esketamine on Postoperative Depression in Patients Undergoing Bariatric Surgery With Preoperative Depression	NCT07179913	The aim of the study is to evaluate the antidepressant effects of low-dose esketamine in obese patients with preoperative depression who are scheduled for bariatric surgery.	Phase 4
Low-dose S-Ketamine and Postpartum Depression in Parturients With Prenatal Depression	NCT03927378	Since prenatal depression is a major risk factor for postpartum depression, this study investigates whether low-dose postnatal S-ketamine infusion may help reduce the risk. Clinical observations indicate stronger analgesic effects, improved anesthesia, and a lower incidence of adverse psychological reactions.	Not Applicable
Ketamine for Depression and Alcohol Dependence	NCT01551329	The study aims to evaluate the effectiveness of ketamine in reducing depressive symptoms in individuals with comorbid major depressive episodes and alcohol dependence.	Phase 1
An Expanded Access Protocol for Esketamine Treatment in Participants With Treatment-Resistant Depression (TRD) Who Do Not Have Other Treatment Alternatives	NCT04476446	The purpose of this study is to provide expanded access to esketamine treatment and to collect additional safety and quality-of-life data until the drug becomes commercially accessible for individuals with treatment-resistant depression (TRD).	Phase 3
A Study of Ketamine Infusions to Treat Clinically Depressed ICU Patients	NCT05803551	The study aims to evaluate the effect of intravenous ketamine infusions in treating ICU patients presenting with depressive symptoms.	Phase 2
Ketamine for Older Adults Pilot	NCT04504175	A pilot study designed to assess the safety and feasibility of intravenous ketamine administration in older adults with treatment-resistant depression (TRD).	Phase 4
Metabolic Profiling of Esketamine Treatment in Depressive Disorder	NCT07002684	Using an untargeted metabolomics approach, this study aims to identify biochemical pathway changes associated with treatment-resistant major depressive disorder (MDD) and changes linked to the mechanism of action of esketamine.	Not Applicable
Biomarkers of Response to Ketamine in Depression: MRI and Blood Assays Before and After Open-Label Intranasal Ketamine	NCT04216888	This pilot study aims to identify predictors of response to intranasal ketamine treatment in patients with treatment-resistant depression using MRI and blood-based biomarkers.	Phase 2/3

**Table 3:** Selected clinical trials investigating the use of psilocybin in the treatment of depression.

Study Title	NCT Number	Brief Description	Phase
Psilocybin Intervention for Veterans Overcoming Treatment-Resistant Depression	NCT07226232	This multisite, randomized controlled trial aims to evaluate the effectiveness and risks associated with psilocybin use in treating depression among U.S. military veterans.	Phase 3
Evaluating the Role of Psilocybin Monitors in Psilocybin Therapy for Treatment-Resistant Depression	NCT07211438	The aim of this study is to investigate how facilitators conducting psilocybin therapy sessions influence treatment outcomes in adults with treatment-resistant depression.	Phase 2
Psilocybin Therapy for Depression in Parkinson’s Disease	NCT06455293	This study seeks to determine whether psilocybin administration improves depressive symptoms in individuals with comorbid Parkinson’s disease and depression.	Phase 2
The Effects of Psilocybin on Self-Focus and Self-Related Processing in Major Depressive Disorder	NCT06247839	This open-label study uses functional MRI to assess rumination and related neural activity in individuals with major depressive disorder after a single dose of psilocybin.	Phase 3
Psilocybin for Treatment-Resistant Depression in Autism With Pre-Post Brain and Cognitive Measurement to Understand Mechanisms	NCT06731621	This open-label clinical study aims to evaluate the feasibility, tolerability, and safety of psilocybin administration in adults with autism and treatment-resistant depression.	Phase 1

**Table 4:** A compilation of all currently available clinical trials listed on ClinicalTrials.gov investigating dextromethorphan–bupropion and its relationship with depression.

Study Title	NCT Number	Short Description	Phase
Efficacy of Dextromethorphan-Bupropion Versus SSRIs in the Treatment of Major Depressive Disorder	NCT06957223	The aim of this study is to compare the antidepressant efficacy of dextromethorphan–bupropion with standard SSRI therapy in adult patients with major depressive disorder.	Not Applicable
Open-Label Safety Study of AXS-05 in Subjects With TRD (EVOLVE)	NCT04634669	A multicenter study designed to evaluate the long-term safety and effectiveness of AXS-05 in patients with treatment-resistant depression (TRD) and major depressive disorder (MDD).	Phase 2
A Trial of Dextromethorphan and Bupropion Sustained-Release Tablets in Patients With Major Depressive Disorder	NCT06958692	A multicenter, randomized, double-blind, placebo-controlled trial assessing the efficacy and safety of sustained-release dextromethorphan–bupropion tablets in adult Chinese patients with major depressive disorder.	Phase 3
Open-Label Safety Study of AXS-05 in Subjects With Depression	NCT04039022	An open-label, long-term safety study of AXS-05 in patients with major depressive disorder (MDD), including those with treatment-resistant depression.	Phase 3
Assessing Symptomatic Clinical Episodes in Depression	NCT03595579	A randomized, double-blind, active-controlled study evaluating AXS-05 for the treatment of MDD.	Phase 2
A Study to Evaluate the Efficacy of AXS-05 Compared to Bupropion in Preventing the Relapse of Depressive Symptoms	NCT06223880	A randomized, double-blind, active-controlled, multicenter trial assessing the efficacy of AXS-05 compared with bupropion in preventing relapse of depressive symptoms in MDD patients who initially responded to AXS-05.	Phase 4
A Trial of AXS-05 in Patients With Major Depressive Disorder	NCT04019704	A randomized, double-blind, placebo-controlled, multicenter trial investigating AXS-05 in patients with major depressive disorder.	Phase 3
Drug Metabolism and Antidepressant	NCT02438072	A systematic investigation of the relationship between drug-metabolizing enzyme activity (phenotypically assessed), antidepressant plasma concentrations, clinical effectiveness, and tolerability.	Not Applicable
Mechanistic Evaluation of Response in TRD (MERIT)	NCT04608396	An evaluation of relapse prevention with AXS-05 compared with placebo in patients with treatment-resistant depression.	Phase 2

**Table 5:** Selected clinical studies from the ClinicalTrials.gov database investigating zuranolone in the treatment of depression.

Study Title	NCT Number	Brief Description	Phase
Allopregnanolone (Zuranolone) in Post-stroke Depression	NCT06759558	The aim of this study is to determine whether zuranolone can be safely used in individuals who have experienced a stroke and who present with moderate or severe post-stroke depression, and whether it helps alleviate depressive symptoms.	Phase II
A Study to Learn More About How Zuranolone Affects Postpartum Depression Symptoms in Participants Who Took it Within 1 Year After the End of Their Pregnancy	NCT07047820	The primary objective of this study is to evaluate the effectiveness of zuranolone in reducing symptoms of postpartum depression (PPD), measured using the Edinburgh Postnatal Depression Scale (EPDS) on Day 15.	Not Applicable
Zuranolone Pharmacokinetics (PK) and Safety Study in Adolescent Participants With Major Depressive Disorder (MDD)	NCT05655507	This study aims to assess the pharmacokinetics and safety of zuranolone in adolescents (aged 12–17 years) diagnosed with unipolar major depressive disorder.	Phase 1

**Table 6:** Selected clinical studies from the ClinicalTrials.gov database investigating brexanolone in the treatment of depression.

Study Title	NCT Number	Brief Description	Phase
Study on Allopregnanolone and Depression in Perimenopausal Women	NCT05329779	The aim of this study is to investigate how allopregnanolone, a metabolite of progesterone, influences behavior and neurobiology that may underlie perimenopausal depression.	Phase 4
A Study to Assess the Safety of Brexanolone in the Treatment of Adolescent Female Participants With Postpartum Depression (PPD)	NCT03665038	This multicenter study evaluated the safety, tolerability, and pharmacokinetics of brexanolone in adolescent participants diagnosed with postpartum depression.	Phase 3
A Study to Evaluate Multimodal Neuroimaging Parameters in Women With Postpartum Depression Who Are Receiving ZULRESSO™ (Brexanolone)	NCT04273191	This study aims to assess the relationship between changes in depressive symptoms and alterations in neuroimaging parameters in women receiving ZULRESSO™ (brexanolone).	Phase 4

**Table 7:** Selected clinical studies from the ClinicalTrials.gov database investigating cariprazine in the treatment of depression.

Study Title	NCT Number	Brief Description	Phase
A Study to Assess Change in Disease Activity and Adverse Events (AEs) With Cariprazine in the Treatment of Depressive Episodes in Pediatric Participants (10 to 17 Years of Age) With Bipolar I Disorder	NCT04777357	The aim of this study is to evaluate the efficacy and safety of cariprazine in treating depressive episodes in pediatric patients with bipolar I disorder (ages 10–17).	Phase 3
Dopamine D3 Receptor Occupancy in Bipolar Depression	NCT05060549	This study aims to understand the detailed mechanisms of cariprazine’s action in bipolar depression using positron emission tomography (PET).	Phase 4
Lithium Versus Cariprazine in the Acute Phase Treatment of Bipolar Depression (DUAG9)	NCT05913947	The study aims to compare the effects of lithium and cariprazine in the acute treatment of depressive episodes in bipolar disorder.	Phase 4
Effectiveness of Cariprazine Monotherapy for Treatment of Major Depressive Disorder	NCT05933538	This study aims to evaluate the efficacy and safety of cariprazine monotherapy compared with standard treatment for major depressive disorder (MDD).	Phase 4

**Table 8:** Overview of novel antidepressant agents used in the treatment of depression, including their primary mechanisms of action, synaptic effects, and downstream mechanisms.

Drug	Mechanism of Action	Synaptic Effect	Downstream Effects	References
Ketamine	Non-competitive antagonism of NMDA receptors, primarily on GABAergic interneurons, leading to disinhibition of glutamatergic neurons and a transient increase in glutamatergic signaling with preferential AMPA receptor activation	Rapid enhancement of AMPA receptor–dependent transmission, increased number and stability of synaptic connections, and increased incorporation of Ca^2+^-permeable AMPA receptors into the postsynaptic membrane	Activation of the BDNF–TrkB axis with engagement of PI3K–AKT–mTORC1 and ERK/MAPK cascades, relief of translational repression of BDNF via eEF2K deactivation, and enhanced synaptogenesis with long-term reorganization of neuronal networks	[42,43,44,45,46,47,48,49,50,51]
Psilocybin	Agonism of serotonergic 5-HT_2_A receptors with hallucinogen-specific signaling via Gq/11 and Gi/o proteins, and functional coupling of 5-HT_2_A receptors with mGlu_2_	Increased activity of prefrontal cortical pyramidal neurons, enhanced glutamatergic transmission, and secondary activation of AMPA and NMDA receptors facilitating synaptic reorganization	Induction of early response genes related to synaptic plasticity, activation of the BDNF–TrkB axis and PI3K–AKT, Ras/MAPK, and PLCγ pathways, modulation of default mode network activity, and immunomodulatory effects via attenuation of pro-inflammatory signaling	[52,53,54,55,56]
Zuranolone	Positive allosteric modulation of GABA-A receptors at α and β subunits, affecting both synaptic and extrasynaptic receptors	Enhancement of phasic and tonic GABAergic inhibition, neuronal hyperpolarization, and rapid stabilization of excitatory–inhibitory balance within cortico-limbic circuits	Normalization of Ca^2+^-dependent signaling pathways, secondary reduction in excessive glutamatergic activity, and modulation of gene expression associated with stress responses and synaptic plasticity	[57,58,59,60]
Brexanolone	Positive allosteric modulation of GABA-A receptors by a neuroactive steroid identical to endogenous allopregnanolone	Potentiation of both synaptic and extrasynaptic GABAergic inhibition, resulting in reduced neuronal excitability and normalization of mood-regulating circuit activity	Limitation of pathological Ca^2+^ influx, normalization of Ca^2+^-dependent signaling pathways, and stabilization of gene expression involved in plasticity and stress adaptation, particularly in the context of postpartum neurosteroid dysregulation	[61,62,63,64]
Dextromethorphan–bupropion	NMDA receptor antagonism and sigma-1 receptor agonism by dextromethorphan, combined with CYP2D6 inhibition and modulation of noradrenergic and dopaminergic neurotransmission by bupropion	Modulation of glutamatergic transmission with a shift toward AMPA receptor–dependent signaling and improved synaptic function in motivational and affective circuits	Activation of the BDNF–TrkB axis and PI3K–AKT–mTOR and MAPK/ERK pathways, sigma-1 receptor–mediated neuroprotection, and sustained enhancement of synaptic plasticity in the prefrontal cortex and limbic structures	[65,66,67,68,69]
Gepirone ER	Selective agonism of serotonergic 5-HT_1_A receptors with initial activation of presynaptic autoreceptors followed by secondary desensitization during chronic treatment	Gradual enhancement of serotonergic transmission in limbic and cortical regions, leading to improved emotional regulation and reduction in anxiety symptoms	Activation of cAMP/PKA, PI3K/AKT, and MAPK/ERK pathways, modulation of gene expression related to neuroadaptation, neurogenesis, and neuronal survival, and long-term normalization of emotion-processing networks	[70,71,72,73,74,75,76]
Cariprazine	Partial agonism of dopamine D3 and D2 receptors with marked preferential affinity for D3 receptors, and partial agonism of 5-HT_1_A receptors	Stabilization of dopaminergic signaling within mesolimbic and mesocortical pathways, with improvement in reward processing, motivation, and psychomotor drive	Modulation of Gi/o-dependent signaling pathways, including PKA, PI3K/AKT, and MAPK/ERK, regulation of gene expression related to synaptic plasticity and mitochondrial function, and long-term normalization of motivational circuits	[77,78,79,80,81,82]
(R)-ketamine	AMPA receptor activation and induction of the BDNF–TrkB axis independent of sustained NMDA receptor blockade, with a significant contribution of microglial TGF-β1 signaling	Restoration of normal synaptic plasticity in the prefrontal cortex and hippocampus, increased dendritic spine density, and stabilization of synapses	Activation of ERK–CREB–BDNF signaling in neurons and microglia, involvement of the ERK–NRBP1–CREB–BDNF axis, and durable structural and functional remodeling of limbic–cortical circuits	[83,84,85,86,87,88]

## Data Availability

No new data were created or analyzed in this study. Data sharing is not applicable to this article.
